# Differences in neuronal ciliation rate and ciliary content revealed by systematic imaging-based analysis of hiPSC-derived models across protocols

**DOI:** 10.3389/fcell.2025.1516596

**Published:** 2025-04-11

**Authors:** Walther Haenseler, Melanie Eschment, Beth Evans, Marta Brasili, Joana Figueiro-Silva, Fee Roethlisberger, Affef Abidi, Darcie Jackson, Martin Müller, Sally A. Cowley, Ruxandra Bachmann-Gagescu

**Affiliations:** ^1^ URPP Adaptive Brain Circuits in Development and Learning, University of Zurich, Zurich, Switzerland; ^2^ Department of Molecular Life Sciences, University of Zurich, Zurich, Switzerland; ^3^ Clinical Research Priority Program Praeclare, University of Zurich, Zurich, Switzerland; ^4^ Institute of Medical Genetics, University of Zurich, Schlieren, Switzerland; ^5^ FHNW School of Life Sciences, University of Applied Sciences and Arts Northwestern Switzerland, Muttenz, Switzerland; ^6^ Institute for Regenerative Medicine, University of Zurich, Schlieren, Switzerland; ^7^ James and Lillian Martin Centre for Stem Cell Research, Sir William Dunn School of Pathology, University of Oxford, Oxford, United Kingdom

**Keywords:** cilia, human iPSC (induced pluripotent stem cells), neurons, ciliopathies, immunofluorescence staining

## Abstract

**Introduction:**

Ciliopathies are a group of human Mendelian disorders caused by dysfunction of primary cilia, small quasi-ubiquitous sensory organelles. Patients suffering from ciliopathies often display prominent neurodevelopmental phenotypes, underscoring the importance of primary cilia during development and for function of the central nervous system (CNS). Human tissues, in particular from the CNS, are very hard to obtain for research. Patient derived- or genetically engineered human induced pluripotent stem cells (hiPSCs) are therefore a precious resource for investigating the role of cilia in human neurons.

**Methods:**

In this study we used a variety of 2D and 3D neuronal differentiation protocols in multiple hiPSC lines and systematically analyzed ciliation rates and ciliary length in hiPSCs, neural stem cells (NSCs), immature and different types of mature neurons using immunofluorescence.

**Results:**

We found that ciliation rate varied substantially between cell lines and differentiation protocols. Moreover, ciliation rate depended on differentiation stage, being maximal in NSCs and decreasing with neuronal maturation. In various types of mature neurons obtained with different protocols, we found ciliation rates to be as low as ∼10%. Neuronal density also played an important role, with higher ciliation in denser cultures. We further investigated the ciliary protein content in these cells at different differentiation stages using commonly used antibodies against ARL13B, INPP5E, AC3 and GPR161. Cilia in hiPSCs, NSCs and neurons were all positive for ARL13B, with a decreasing trend in intensity in more mature neurons. Likewise, INPP5E was present in all cilia analyzed, while AC3 positivity increased as maturation proceeded. Interestingly, we found that while GPR161 signal almost completely disappeared from cilia upon Sonic hedgehog (SHH) stimulation in NSCs and immature neurons, this was not the case in more mature neurons, suggesting a possible developmental time window for cilia-dependent SHH signaling.

**Conclusion:**

Taken together, our results provide a systematic description of cilia in hiPSC-derived neuronal cells generated with different protocols, underscoring the importance of selecting the optimal model system and controls for investigating primary cilia in hiPSC-derived neuronal cells.

## Introduction

**FIGURE 1:**
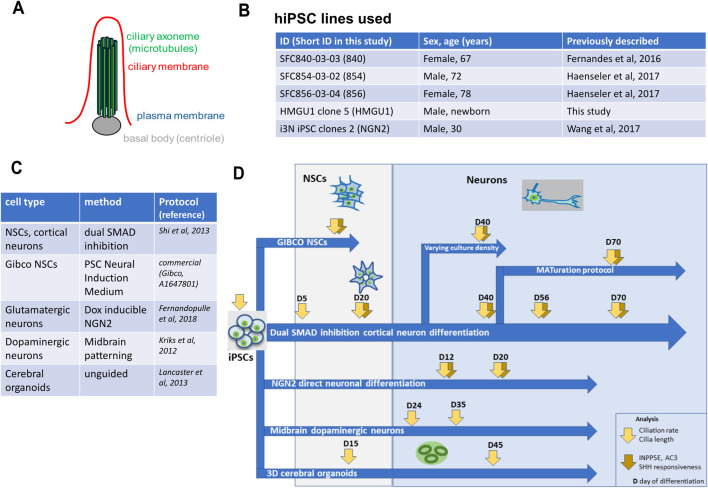
Overview of cell lines, protocols and analyses performed in this study. **(A)** Schematic of the basic structure of a primary cilium showing the basal body in grey, the micro-tubule-based axoneme in green and the ciliary membrane in red. **(B)** Overview of the hiPSC lines used in this study. **(C)** Overview of the hiPSC differentiation protocols used in this study. **(D)** Overview of the experimental flow performed in this work. hiPSC were differentiated with different neuronal protocols and analysis of ciliation rate, cilia length, cilia content (ARL13B, INPP5E, AC3) and cilia function (GPR161 depletion from cilia upon SHH stimulation) was performed at different timepoints.

The primary cilium is a sensory organelle protruding from most mammalian cells. Its core is formed by microtubules (MT) forming the ciliary axoneme, that protrudes from basal bodies (modified centrioles) and is surrounded by the ciliary membrane, a specialized compartment of the plasma membrane (Figure 1A) (Sorokin, 1968; Satir et al., 2010). Patients with defects in primary cilia suffer from ciliopathies and often exhibit prominent neurodevelopmental phenotypes, including structural and functional abnormalities (encephalocele, midbrain and hindbrain malformations, nodular heterotopias, ventriculomegaly, intellectual disability, seizures, etc.) (Bachmann-Gagescu et al., 2014; Bachmann-Gagescu et al., 2015). Currently the bulk of our knowledge on the role of cilia in the central nervous system (CNS) comes from clinical data, animal models or cell lines. Research with patients is restricted to clinical data and non-invasive imaging methods such as MRI, since *post mortem* samples are rare and typically come with comorbidities while investigation of the prenatal aspects of CNS development is possible only through imaging with ultrasound/ MRI or pregnancy loss or termination. Patient-derived induced pluripotent stem cells (hiPSCs) and the plethora of neuronal differentiation protocols recently developed offer unique opportunities to investigate direct consequences of ciliary defects early in neuronal development, and upon further maturation of human neurons. With this manuscript we want to share our experience with imaging of primary cilia in different hiPSC-derived neuronal differentiation protocols from hiPSC, neural stem cells (NSCs), young and matured neurons in different hiPSC lines.

Cilia on neurons *in vivo* have been described on the cell soma, as confirmed by a recent study using serial EM reconstruction of human cortical volume showing that neurons in human brain are ciliated and that their cilium changes depending on the type of neuron (Wu et al., 2024). The role of ciliopathy proteins is typically investigated by analyzing ciliation rate and ciliary length in mutant cells compared to wild-type control cells. Primary cilia are generally analyzed using immunofluorescence, relying on a set of antibodies against acetylated alpha-tubulin to highlight the axonemal microtubules or against ADP-ribosylation factor-like 13B (ARL13B), adenylyl cyclase 3 (AC3), Inositol polyphosphate-5-phosphatase E (INPP5E) or the G-protein-coupled receptor GPR161 which mark the ciliary membrane. Which of these markers are useful in hiPSC-derived neuronal models has not been systematically assessed yet.

Anti-acetylated tubulin antibody stains all axons as well as cilia and is therefore not so useful in neuronal cells to analyze cilia (Berbari et al., 2007). Work in mouse has shown that in the mature brain ARL13B predominantly marks cilia on astrocytes, while neuronal cilia are rather positive for AC3 (Bishop et al., 2007; Brewer et al., 2024). In contrast, hiPSC-derived hypothalamic arcuate-like neurons are positive for ARL13B and AC3 (Wang et al., 2021). INPP5E is expected to localize to the ciliary membrane of all primary cilia (Zhang et al., 2022) while GPR161, a negative regulator of Hedgehog signaling, localizes to the ciliary membrane dynamically and also to the recycling endocytic compartment (Mukhopadhyay et al., 2013). Similar as in other cell types, it was previously shown that GPR161 disappears from cilia upon SHH stimulation in hiPSC-derived neuronal progenitor cells (NPC) isolated from neuronal rosettes (Boutaud et al., 2022). Hence, GPR161 disappearance from cilia upon SHH pathway stimulation can be used as a functional assay of cilia.

Given the increasing interest in cilia-related research using hiPSC-derived neuronal models, we sought to perform a systematic analysis comparing different control lines and differentiation protocols (Figure 1). We found that ciliation rate is highly variable in hiPSCs but higher and more consistent in neural stem cells (NSCs) across cell lines and differentiation protocols. Ciliation rate substantially decreased in neurons as maturation proceeded and was directly correlated to density of the neuronal cell culture. Cilia on neurons remained positive for ARL13B up to day 70 (last timepoint assessed) but with decreasing intensity, while INPP5E and AC3 were both good markers for neuronal cilia. Ciliary GPR161 positivity decreased after SHH stimulation as expected in NSCs but not in matured neurons, suggesting that the responsiveness of cilia to Hedgehog signaling decreases in these neuronal cultures.

## Materials and methods

### hiPSC line culture

hiPSC lines used in this work were derived from fibroblasts obtained from healthy individuals after informed consent and were previously published (Fernandes et al., 2016; Kunze et al., 2018; Haenseler et al., 2017; Wang et al., 2017). Experiments followed international guidelines and local regulations. Details on the four hiPSC lines used in this study are summarized in the table shown in Figure 1B.

Derivation of the hiPSC lines SFC840-03-03 (840), SFC854-03-02 (854) and SFC856-03-04 (856), from fibroblasts of healthy donors was described previously (Haenseler et al., 2017; Fernandes et al., 2016). These hiPSC lines are also distributed by EBISC as STBCi026-A/RRID:CVCL_RB85 (hereafter referred to as “840”), STBCi066-A/RRID:CVCL_RC86 (hereafter referred to as “854”), and STBCi063-A/RRID:CVCL_RC81 (hereafter referred to as “856”). We maintained the 840, 854 and 856 hiPSCs in E8 medium on Geltrex-coated tissue culture treated vessels and passaged as small clusters of cells by splitting with 0.5 mM EDTA as described previously (Beers et al., 2012). We produced masterbatches of these hiPSCs, that we quality controlled, including SNP array for genome integrity, and started the differentiations of our experiments below five passages from thawing the hiPSCs.

We subcloned the previously described hiPSC line HMGU1 (alternative name ISFi001-A/RRID:CVCL_YT30) (Kunze et al., 2018) and worked with HMGU1 subclone 5. The hiPSC line HMGU1 subclone 5 was quality controlled and expanded on Geltrex-coated vessels by EDTA passage in different media depending on the differentiation protocol used: for cortical neuron differentiation in Essential8 (E8) medium (Thermo Fisher), for Gibco NSCs and cerebral organoids in StemFlex medium (Thermo Fisher) and for dopaminergic neurons in StemMACS iPSC Brew (Miltenyi Biotec).

We also worked with i3N hiPSC line NWTC11.G3-WT that has a doxycycline-inducible neurogenin 2 (NGN2) cassette stably inserted in the WTC11 hiPSC line (Wang et al., 2017), (hereafter referred to as “NGN2”). This line was maintained in E8 medium and quality controlled with a SNP array. The SNP arrays show no major chromosomal abnormalities in any of the lines (Supplementary Figures S1–S5).

### hiPSC neuronal differentiations

The different differentiation protocols are summarized in the table shown in Figure 1C. Cortical neurons, cerebral organoids, dopaminergic neurons and i3 Neurons (NGN2) were differentiated following published protocols (Shi et al., 2012; Kriks et al., 2011; Lancaster and Knoblich, 2014; Fernandopulle et al., 2018). We also produced neuronal stem cells with the commercial PSC Neural Induction Medium (A1647801) from Gibco (“Gibco NSCs” hereafter). We further combined previously described methods to obtain uniform, mature low density neurons by applying a cocktail of proliferation inhibitors and maturation factors to cortical neurons from day 45 onwards (Kemp et al., 2016; Qi et al., 2017). All detailed differentiation protocols including all reagents used are provided in the Supplementary Material.

### Sample preparation and immunofluorescent stainings

We applied standard fixation and immunofluorescent staining methods; all details for sample preparation and staining including antibodies and reagents used are provided in the Supplementary Material. Organoids were cryosectioned before staining (details in the Supplementary Material).

### Microscopy and image analysis for cilia counting and 3D reconstruction for ciliary length measurement

Microscopes used included an SP8 inverse confocal laser scanning microscope (Leica) [z-stacks with 0.3 µm step sizes with a ×63 oil objective (HC PL APO CS2, Leica)] or a BC43 spinning disc confocal (Andor) [0.3 µm step size, with a ×63 oil immersion objective (CFI Plan Apochromat Lambda D 60X Oil)].

For assessment of ciliation rate, we manually counted cilia on maximum intensity z-projected confocal images using the Fiji cell counter plugin. We counted nuclei, pericentrin (PCNT)+ basal bodies (centriole pairs were counted as one basal body) and ARL13B+ cilia (=ARL13B signal that protrudes from a PCTN+ basal body). We also counted INPP5E+, AC3+ and GPR161+ cilia (=INPP5E, AC3 or GPR161 signal that proceeds from a PCNT+ basal body). Given that in some conditions, it is difficult to attribute individual cilia to specific neurons/nuclei, we chose to quantify ciliation rate as the proportion of cilia over basal bodies. Ciliation rates are indicated as mean of a sample ± standard deviation, with n = number of samples unless stated otherwise, where one sample represents an individual cell culture well stained.

For measurement of ciliary length, we used the ImageJ plugin CiliaQ for 3D reconstruction of cilia and extracted ciliary length and orientation in z-direction from the reconstructed data in CiliaQ (Hansen et al., 2021). Image processing with CiliaQ was applied to high resolution images acquired with an SP8 confocal microscope in the ARL13B channel. Cilia length represents the length of the ARL13B signal. We manually checked through the reconstruction data and only kept the reconstructed ARL13B+ objects that had a PCNT+ basal body. Cilia length is indicated as mean ± standard deviation, with n = number of cilia.

Cerebral organoid cryosections stained for ciliary markers were imaged using a Leica SP8 confocal microscope (at 63x with 7.5 optical zoom). We took images every 0.2 μm in the z-plane. Z-stacks from at least three ventricles of three different organoids in three independent organoid differentiations were analyzed (= at least nine images). Images were deconvoluted using Huygens Software and subsequently analyzed using the ImageJ/Fiji plug-in CiliaQ. The channel for ciliary length measurement (ARL13B staining) was used for segmentation and subsequent length assessment. The segmentation method ReyniEntropy was applied, with a Gaussian blur of 1.5. Cilia touching X/Y/Z borders of the image were excluded.

For analysis of additional markers (INPP5E, AC3), we assessed ciliation rate of ARL13B-positive cilia and ciliation rate of the additional ciliary marker separately and from these numbers calculated the percentage of INPP5E-positive cilia over ARL13B-positive cilia respectively AC3-positive cilia over ARL13B-positive cilia.

### Statistical analysis

GraphPad Prism9 was used for data visualization and statistical analysis. The statistical test applied is indicated in the figure legends and/or the main manuscript. In the graphs for ciliation rates we displayed all Fields of View (FOVs) as single datapoints to illustrate the observed variability, but for statistical analysis we used the mean of the FOVs acquired for each individual sample unless stated otherwise. One sample is an individual immunofluorescence staining (of one individual cell culture well).

## Results

### Primary cilia in undifferentiated hiPSC are short and ciliation rate is highly variable

**FIGURE 2:**
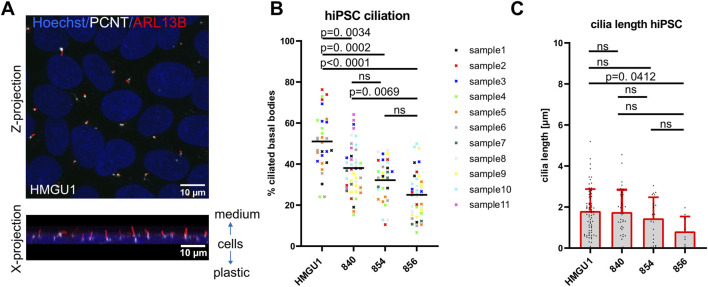
Variability in ciliation rate in different undifferentiated hiPSC lines. **(A)** Confocal image of primary cilia in hiPSCs as a maximum intensity z-projection (top) and x-projection (bottom) showing that cilia sit on top of cells pointing towards the medium. **(B)** Scatter plot showing ciliation rate (% PCNT-positive basal bodies colocalizing with ARL13B-positive cilia) of different sub-confluent iPSC lines at day 2 after replating. Each datapoint represents one fields of view (FOV), with the color-coding indicating different samples (individual wells) for each of the four cell lines. Black lines show the mean for each hiPSC line. Statistical analysis was performed with a Tukey’s multiple comparison test using the mean ciliation rate of each sample. Note the variability between lines, individual samples and between the ROIs of each sample. **(C)** Cilia length measured in 3D with CiliaQ. Each datapoint represents a single cilium. Plots show the mean and standard deviation.

Before starting differentiation into neuronal cells, we first wanted to assess primary cilia in human induced pluripotent stem cells (hiPSCs). These are cycling cells and actively dividing cells are expected to lack a cilium during the division process. To assess the ciliation rate of human hiPSCs we performed immunofluorescence using antibodies against the ciliary membrane-associated protein ARL13B and against the centriolar protein pericentrin (PCNT) in four different hiPSC lines derived from healthy individuals (Figure 1B and 2A). We found that ciliation rate, as determined by manual count of ARL13B-positive signal correlating with PCNT-positive signal, was highly variable between different hiPSC lines and between independent experiments, with an average ciliation rate of 34.43 ± 12.21% across all samples [n = 37 samples; ± standard deviation (SD)] (Figure 2B). Interestingly, ciliation rate was not influenced by the proliferative status since we found no significant differences between lines when assessing proliferation by quantifying cells in S-phase using EdU or M-phase based on Hoechst staining, despite different ciliation rates (Supplementary Figure S6). Cilia on hiPSCs were short with an average length of 1.677 ± 1.071 µm (n = 138 cilia; average cilia length ± SD) (Figure 2C). We further determined that cilia were aligned towards the cell culture medium and away from the plastic (Figure 2A and Supplementary Figure S7).

#### High ciliation rate in hiPSC-derived neural stem cells

**FIGURE 3:**
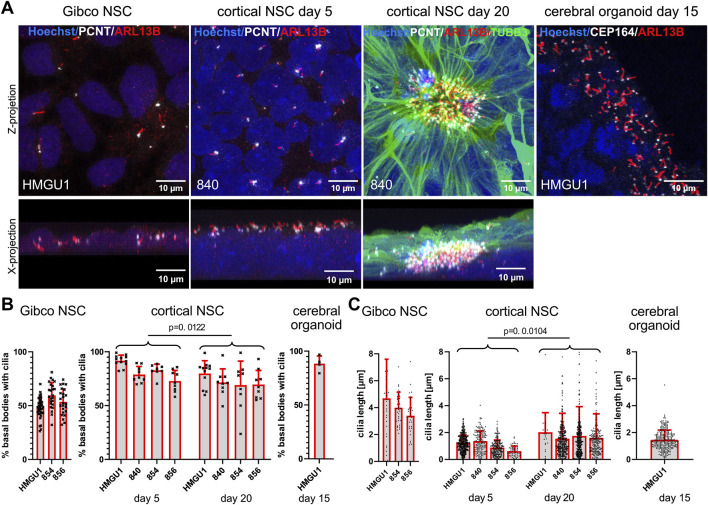
High ciliation rate in hiPSC-derived neural stem cells generated with different protocols. **(A)** Immunofluorescence maximum projection confocal images of neural stem cells (NSCs) produced with the Gibco kit (“Gibco NSCs”), NSCs produced with the dual SMAD inhibition cortical neuron differentiation protocol at day 5 and day 20 after induction (“cortical NSCs”) and NSCs in cerebral organoids on day 15 after induction. Cilia are marked with anti-ARL13B antibody (red) and basal bodies with anti-PCNT or anti-CEP164 antibody (white) as indicated. Top images are z-projections and bottom images are x-projections (only for the adherent cells). The cerebral organoid image (far right) shows a zoomed in region at the ventricular lumen of the organoid. **(B)** Quantification of ciliation rate: cilia were counted as ARL13B-positive signal (red) colocalizing with PCTN-positive basal bodies for the adherent cells or with CEP164-positive basal bodies in cerebral organoids (white). Each data point represents one field of view (FOV). Comparisons between day 5 and day 20 cortical NSCs were performed using an unpaired students t-test using the mean ciliation rate for each sample (see methods). Note the high ciliation rate across lines in cortical NSCs and in organoids and the somewhat lower ciliation rates in Gibco NSCs. **(C)** Quantification of cilium length determined using CiliaQ. Each data point represents one cilium. Comparisons between day 5 and day 20 cortical NSCs were performed using an unpaired students t-test using all datapoints from individual cilia **(C)**. Note that ciliary length increases slightly during differentiation, while ciliation rates slightly decrease. Cilia length is highest in Gibco NSCs. Bars show the mean and SD for each hiPSC line.

We next differentiated hiPSCs to neurons following various neuronal differentiation protocols (Figure 1D and 3A) and first assayed ciliation at neural stem cell (NSC) state. We used a commercial kit to generate expandable neural stem cells [“Gibco NSCs” (Yan et al., 2013)], a dual SMAD inhibition protocol producing NSCs (based on Shi et al. (2012)) and an undirected 3D cerebral organoids protocol (modified from Lancaster and Knoblich (2014)) with a relatively pure NSC population at day 15.

##### Gibco NSCs

NSCs produced with the Gibco protocol fixed 2 days after final plating when they reached a confluency of around 70% were mostly PAX6 and Nestin-positive (Supplementary Figure S8). In these cells, which do not form rosettes but rather a homogenous sheet of cells, the PCNT-positive basal bodies and ARL13-positive cilia showed no particular orientation and cilia could be counted unambiguously as they did not overlap (Figure 3A, Supplementary Figures S7–S8). The average ciliation rate was 52.47 ± 8.539% (n = 15 samples) (Figure 3B). The average length of cilia on NSCs in this line with the Gibco protocol approached 4 µm (4.036 −/+2.025 µm, n = 98 cilia) (Figure 3C).

##### Dual SMAD inhibition protocol NSCs at day 5

In comparison, using the dual SMAD inhibition protocol to generate NSCs, 5 days after induction from the same lines, we observed that the cells were densely packed and stacked on top of each other. Ciliation rate was higher in all lines and in all differentiation runs with an average ciliation rate of 82.21 −/+8.654% (n = 13 samples). Using this protocol, cilia display a more uniform orientation with their centrioles lying on top of a several nuclei-thick layer and the cilia were facing towards the medium with a positive z orientation (Figures 3A and Supplementary Figure S7). Interestingly, now the cilia were consistently very short with an average length of 1.138 ± 0.6497 µm (n = 956 cilia) (Figure 3C). It is particularly interesting that the same hiPSC lines, differentiated into similar cell types using two different protocols, displayed such differences in ciliary length and ciliation rate.

##### Dual SMAD inhibition protocol NSCs at day 20 (neural rosette-stage)

With further differentiation of these NSCs using the dual SMAD inhibition protocol, we observed PAX6- and Nestin-positive NSCs forming neuronal rosettes, with cilia accumulating in the center of the rosette at day 20 of cortical differentiation (Supplementary Figure S8). Thin and weakly TUBB3-positive filaments from each NSC project to the center of the rosette, carrying a PCNT-positive basal body at the end of this process and the ARL13B-positive cilium further projecting into the lumen of the neuronal rosette (Figure 3A). At this stage, it was often difficult to unambiguously count basal bodies and cilia in the densely packed center of the rosette, as substantial overlap between cilia was observed. Nevertheless, we found an average ciliation rate of 73.03 ± 8.625% (n = 13 samples) in day 20 cortical NSCs (Figure 3B). There was no common orientation of cilia in the center of these rosettes in the z-plane (Supplementary Figure S7) and the average cilium length was 1.898 ± 2.218 µm (n = 1799 cilia), which is significantly longer than at day 5 of the same neuronal induction (p = 0.0104, unpaired students t-test) (Figure 3C). The average length of cilia remains however shorter than in the NSCs generated using the Gibco protocol.

##### NSCs in cerebral organoids at day 15

We next assayed cerebral organoids at day 15, when they consist mainly of PAX6-and Nestin-positive NSCs (Supplementary Figure S8), which project their cilia on the apical side of the cell into the lumen (Figure 3A). We found cilia on most of the basal bodies at the ventricular zone with a ciliation rate of 88.41 ± 7.105% (n = 4 samples) (Figures 3A,B). The average length of cilia in these day 15 cerebral organoids was 1.445 ± 0.6673 µm (n = 346 cilia) (Figure 3C).

In summary, all the NSCs generated by the different 2D and 3D differentiation protocols were generally highly ciliated with a strong ARL13B signal allowing segmentation and 3D reconstructions. NSCs generated with the Gibco protocol are certainly the easiest to assay as the cilia stand alone and are the longest, but these cells display the lowest ciliation rate among NSCs. The complexity of the analysis increases from day 5 to day 20 cortical NSCs and in the 3D cerebral organoids.

### Decreased ciliation rate with increasing neuronal maturity in hiPSC-derived “unguided” cortical neurons

The dual SMAD inhibition NSCs from the four different lines were further propagated to cortical neurons to assess their ciliation rates at day 40, 56 and 70 of differentiation.

**FIGURE 4:**
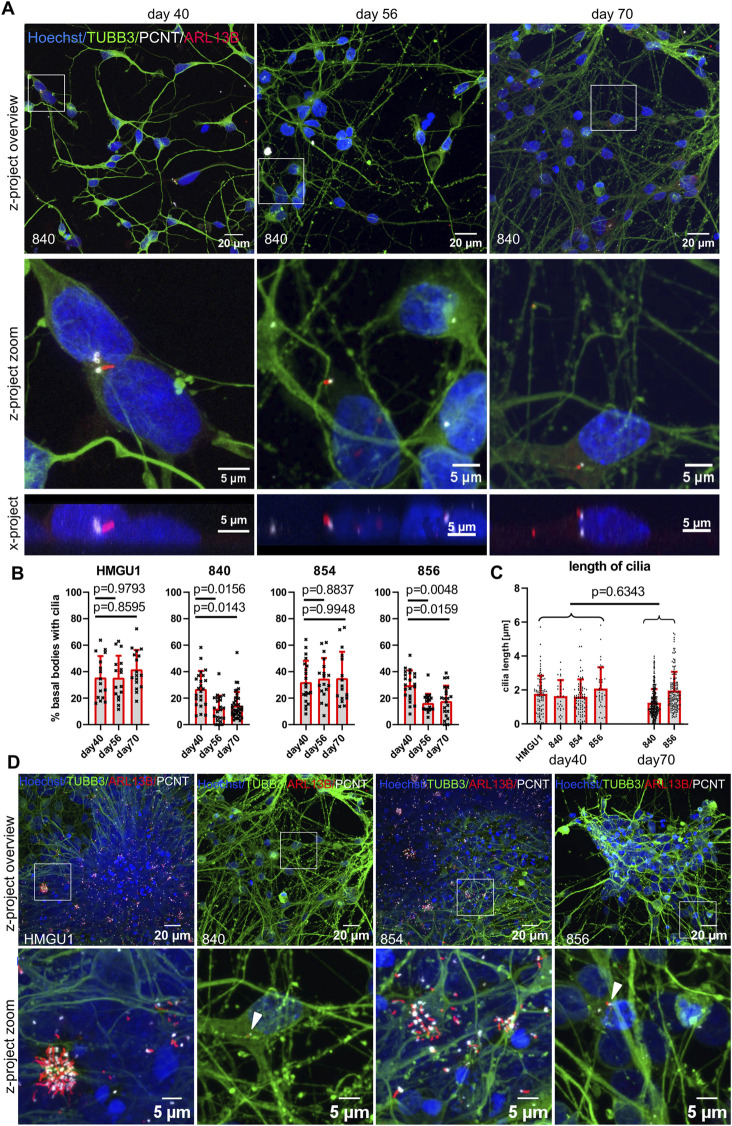
Decreasing ciliation rate in cortical neurons with increasing maturation. Cortical neurons were differentiated as described in the original protocol (Shi et al., 2012) in neuronal maintenance medium. Final plating was performed at day 35 and immunofluorescence was assayed at day 40, day 56 and day 70. **(A)** Representative z-projected confocal images of hiPSC-derived neurons from the 840-line stained with anti-TUBB3 (green) to mark neurons, anti-ARL13B (red) to mark cilia and anti-PCNT (white) to mark basal bodies. The white square indicates the zoomed-in area shown in the panels below. X-projections below show the orientation of the cilia in the y/z- direction: note that the average cilium in neurons is neither facing towards the medium nor towards the tissue culture plastic (also see Supplementary Figure S7). **(B)** Quantification of ciliation rates. Cilia were counted as percent PCNT+ basal bodies with ARL13B+ cilia. Each datapoint represents an individual field of view, displayed with mean and SD. Statistical analysis was performed with a Dunnett’s multiple comparisons test using the mean ciliation rate for each sample. Note the decreasing ciliation rate with maturation in lines 840 and 856 but not HMGU1 and 854. **(C)** Cilia length in 3D was measured with CiliaQ and statistical analysis was performed with an unpaired t-test. Each datapoint represents a single cilium, displayed with mean and SD. **(D)** Overview and zoomed in images of the cultures at day 70 of differentiation from all four lines showing different outcomes, with very dense clumps of cells containing clusters of cilia in lines HMGU1 and 854 but not in lines 840 and 856.

At day 40, cortical neurons from all four hiPSC lines were similarly ciliated with an average ciliation rate of 30.79 ± 13.47% (n = 30 samples) (Figures 4A,B). Cilia had an average length of 1.743 ± 1.080 µm (n = 241 cilia) and were mostly found on the soma of the neurons without a clear orientation in the z plane (Figure 4A and Supplementary Figure S7).

Upon further differentiation, the hiPSC lines 840 and 856 showed a significant reduction of the ciliation rate on day 56 and day 70, but the lines HMGU1 and 854 remained with an unchanged ciliation rate (Figure 4A,B). The length of cilia did not differ as much between lines or between day 40 and day 70, with an average length of 1.501 ± 0.9909 µm at day 70 (n = 454 cilia) (Figure 4C). Importantly, we observed variability in the strength of the ARL13B signal of cilia at day 70 of differentiation with this protocol: while some cilia continued to display a strong ARL13B signal, in many neurons the cilia were only weakly positive for this marker.

When comparing the neuronal cultures generated by the four lines, we noted that despite plating the neurons at the same density at day 35 (20,000 cells/cm^2^), the two lines with higher ciliation rate had much higher cell density. Indeed, at day 56 and day 70 the cultures appeared quite different in the neurons of the different cell lines: in lines HMGU1 and 854 (with stable higher ciliation), cell density was overall higher and the cells tended to cluster in large clumps, while in lines 840 and 856 (with decreasing ciliation) such clusters were not present (Figure 4D). This suggested that the lines yielding cultures with higher density at day 56 and 70 contained residual proliferating cells for a longer period than the lines with lower cellular density. We thus assessed proliferation using EdU and PAX6 to mark progenitor cells, demonstrating as expected a higher proportion of proliferating progenitors at day 40 in lines HMGU1 and 854 (Supplementary Figure S9). The higher ciliation rate in the two proliferating lines (HMGU1 and 854) could thus be explained by a higher proportion of NSCs in the culture and/or by higher density of the resulting neuronal cultures.

To evaluate cell identity of the resulting cultures at later timepoints, we next stained for MAP2 and TUBB3 at day 56 and day 70 which were highly positive in all four cultures, indicating neuronal identity in all four lines (Supplementary Figure S10). We further stained for the forebrain commitment marker FOXG1, which was positive in the majority of cells at day 56 in all four lines, indicating cortical identity (Supplementary Figure S10). Hence, despite persistence of proliferating cells in HMGU1 and 854, all four cultures yielded forebrain neurons at later stages. Interestingly, we observed that the distribution of cilia was particularly high in the regions with clustered neurons, suggesting that cell density plays a role for ciliation of neurons.

Taken together, we find that ciliation rate in these neuronal cultures varies strongly between cell lines, mirroring differences in overall behavior of the line: while starting at day 56 all lines contained cortical neurons, those lines with persistence of proliferating progenitors and subsequent higher culture density (HMGU1 and 854) had higher ciliation rates than the lines with lower culture density (840 and 856). However, ciliation rate was overall lower in more mature neuronal cultures than in NSCs for all lines.

### Effect of cell density and replating on primary cilia

**FIGURE 5:**
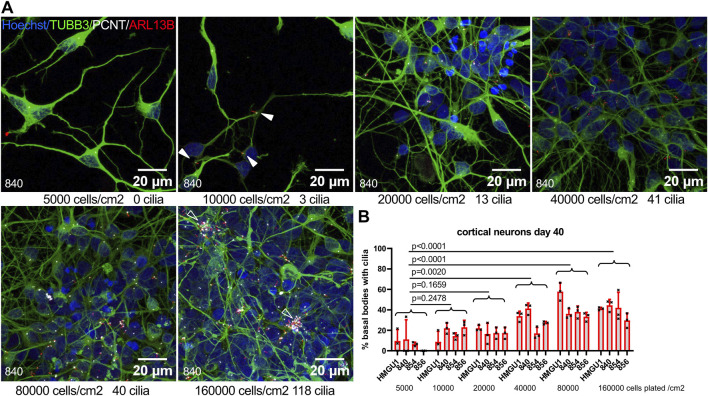
Neuronal ciliation rate correlates with culture density. **(A)** Cortical neurons were plated at different densities at day 35 and fixed at day 40. Representative z-projected confocal images of neurons from the 840 line are shown stained with anti-TUBB3 (green) to mark neurons, anti-ARL13B (red) to mark cilia and anti-PCNT (white) to mark basal bodies. Plating density at day 35 and cilia count in the corresponding field of view (FOV) at day 40 are indicated below each image. Arrowheads indicate sparse cilia. **(B)** Quantification of ciliation rate for all lines at day 40 at different plating densities. Note the increase in ciliation rate with increasing density. Each datapoint represents one FOV. Statistical analysis was performed with a Dunnett’s multiple comparisons test using the mean ciliation rate for each sample. Note cilia clusters at highest culture density (empty arrowheads).

Given this finding that ciliation was much higher on neurons of densely clustered regions compared to sparsely distributed neurons in the same cultures, we next evaluated whether neuronal density influences the ciliation rate. We plated cortical neurons at different densities and assessed ciliation rate at day 40. We found the lowest ciliation rate in cultures plated at the lowest density of 5,000 cells/cm^2^ and significantly higher ciliation rates for densities of 40,000 cells/cm^2^ and above, with a clear direct positive correlation between cell density and ciliation rate (Figure 5). We noted that at the highest neuronal density, accumulations (“clusters”) of cilia were found in all four cell lines.

We also tested if cell density influences the ciliation rate in hiPSC. For this we replated hiPSCs and assayed the samples at day 2 after replating, as in Figure 2, when hiPSCs were ∼50% confluent and had no overlapping nuclei, and at day 3 when hiPSC were over 90% confluent and cells were packed more densely so that nuclei started to overlap. Interestingly, higher hiPSC density led to divergent results depending on the hiPSC line used, with some lines displaying higher ciliation rates in more confluent cultures and others displaying lower ciliation with higher confluency, illustrating again the variability of ciliation rate in hiPSCs (Supplementary Figure S11).

Given that these *in vitro* culture protocols involved replating the maturing cells at given intervals, we also investigated if replating has an influence on the ciliation rate. In the dual SMAD inhibition protocol (Shi et al., 2012), cells are frozen at day 28, then thawed and replated after 7 days (day 35) for final neuronal maturation. For this experiment, we skipped the replating at day 35: we thawed the cells at day 28 directly to the imaging plate and compared these to standard neurons replated at day 35, both being fixed and stained at day 36. We could not find significant changes in ciliation rates between these two conditions (Supplementary Figure S12). We also checked if replating at a later timepoint affects the ciliation rate. For this we compared neurons replated on day 55 to neurons replated at day 35 (standard protocol), both fixed at day 56. Also here, we could not find significant changes in ciliation rate (Supplementary Figure S12).

Taken together, these results indicate that culture density directly impacts ciliation rate in neuronal culture, but not in iPSCs, while replating does not affect ciliation rate.

### Forced maturation of the neuronal cultures results in low ciliation rates

**FIGURE 6:**
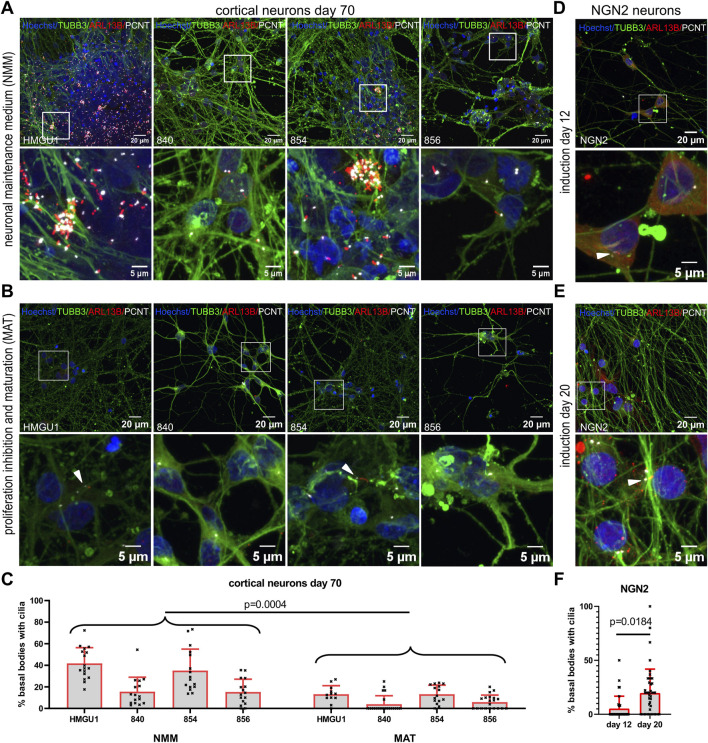
Ciliation rate in neurons across different neuronal differentiation protocols. **(A–C)** Cortical neurons at day 70 of differentiation. From Day 45 onwards neurons were differentiated in standard neuronal maintenance medium (NMM) **(A)** or in medium with proliferation inhibitors and a maturation cocktail (MAT) **(B)**. Representative z-projected confocal images of hiPSC-derived neurons from the 840-line stained with anti-TUBB3 (green) to mark neurons, anti-ARL13B (red) to mark cilia and anti-PCNT (white) to mark basal bodies. **(C)** Quantification of ciliation (as % PCNT+ basal bodies with ARL13B+ cilia) in each line with the two different protocols. Note that the HMGU1 and 854 lines remain more proliferative in NMM (see also Supplementary Figure S9) and display more cilia, while the 840 and 856 lines have less proliferative cells in the same NMM medium. The forced maturation protocol leads to more homogenous cultures across lines with similarly low ciliation rate in all lines. White arrowheads indicate sparse cilia. Also note that the ARL13B signal in the cilia found on the MAT neurons is weaker than on NMM neurons and for many fields of view we found only PCNT+ basal bodies but no ARL13B+ cilia (as in the images displayed in B for lines 840 and 856). **(D and E)** Doxycycline-inducible NGN2 neurons at 12 days **(D)** and 20 days **(E)** after induction, stained similarly with anti TUBB3, ARL13B and PCNT. **(F)** Quantification of the ciliation rate at day 12 or 20 for the NGN2 line. Note an increase in ciliation in this case. Each data point in the plot represents an individual FOV, bars show mean and SD. Statistical analysis was performed with an unpaired t-test using the mean ciliation rate for each sample.

Given the variability observed with the dual SMAD protocol, where cells remain unguided in neural maintenance medium (NMM) (Figure 6A), with variable persistent proliferation, we decided to apply other differentiation protocols. We first modified the dual SMAD inhibition protocol by applying proliferation inhibitors and neuronal maturation factors (MAT) from day 45 of differentiation onwards to obtain more uniform mature neurons. Using this “MAT protocol,” we obtained similar densities of neurons with consistent high levels of MAP2-positive cells and Beta4 spectrin (SPTBN4)-positive axon initial segments at day 70 from all four hiPSC lines (Figure 6B and Supplementary Figure S13). In contrast to the previously applied differentiation in NMM, we no longer observed clusters of cells or of cilia in day 70 neurons in lines HMGU1 and 854, and the four lines produced very similar cultures. Ciliation rate was now significantly lower than in NMM neurons for all hiPSC lines with an average ciliation rate of 9.179 ± 5.587% (n = 15 samples) (Figure 6C). Overall, day 70 MAT neurons had only few ARL13B-positive cilia and mostly with a weak ARL13B signal (Figure 6B).

To compare with a different protocol generating excitatory glutamatergic neurons, we assessed ciliation rate in NGN2 neurons, 12 and 20 days after NGN2 induction (Figures 6D–F). At both timepoints we obtained TUBB3- and MAP2-positive neurons and we found SPTBN4-positive axon initial segments in day 20 NGN2 neurons (Supplementary Figures S13 and S14). Ciliation rate was 5.936 ± 7.177% (n = 5 samples) at day 12 and 19.58 ± 8.338% (n = 6 samples) at day 20 (Figure 6F). Similar to the MAT protocol, the NGN2 neurons only had few ARL13B-positive cilia mostly with weak ARL13B signal.

To evaluate cilia on another type of hiPSC-derived neuron, we differentiated the HMGU1 line to midbrain patterned dopaminergic neurons and assessed ciliation rate at day 24 and 35 of differentiation (Supplementary Figure S15). Overall ciliation rate, when taking all nuclei into account, was 42.86 ± 9.8% (n = 4 samples) at day 24 and significantly reduced to 13.96 ± 5.214% (n = 3 samples) at day 35. Given that these cultures are not composed purely of dopaminergic neurons, we next restricted cilia counts to TH+ dopaminergic neurons and found even lower ciliation rates with 18.94 ± 4.648% (n = 4 samples) at day 24 and 7.032 ± 6.1% (n = 3 samples) at day 35. Length of cilia did not significantly differ between day 24 with 2.301 ± 1.005 µm (n = 28 cilia) and day 35 with 2.210 ± 1.771 µm (n = 48 cilia) (Supplementary Figure S15).

In summary, ciliation rate was overall lower in neurons produced with a variety of protocols than in NSCs.

### Diversity of primary cilia in hiPSC-derived cerebral organoids

**FIGURE 7:**
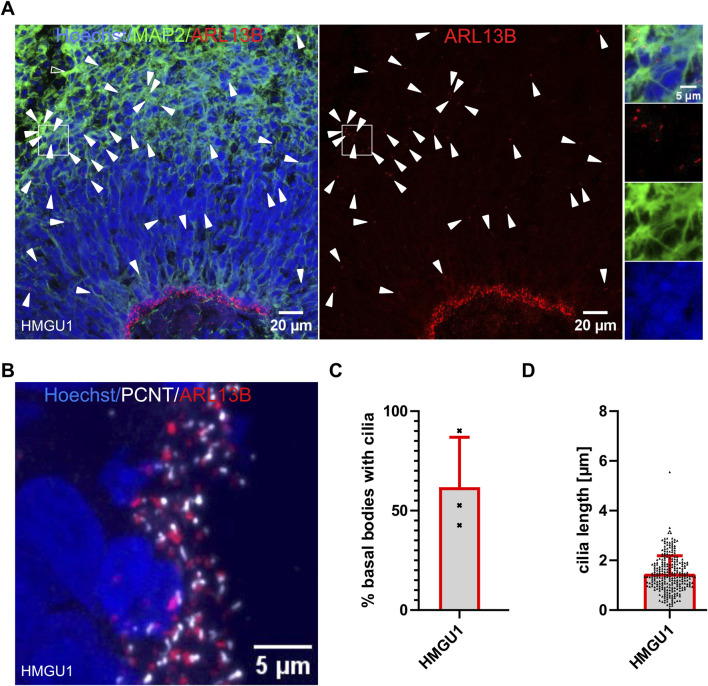
High ciliation rate in progenitors in organoids. **(A and B)** Confocal image of a 45-day-old cerebral organoid cryosection from the HMGU1 line stained with anti-ARL13B to mark cilia and anti-MAP2 to mark neurons. Nuclei are counterstained with Hoechst. **(A)** The lumen of the ventricular zone (VZ) is densely lined by ARL13B+ cilia. Ciliation is sparser at the basal side of the VZ and on mature MAP2+ neurons. Arrowheads indicate cilia not lining the lumen. The boxed area indicates a MAP2-rich region (insets on the right with individual channels showing several ARL13B+ cilia). **(B)** Higher magnification image of the apical-most region at the lumen of the VZ of a cryosectioned 45-day-old organoid stained with anti-ARL13B to mark cilia (red) and anti-PCNT to mark basal bodies (white). Nuclei are counterstained with Hoechst. **(C)** Quantification of ciliation rate at the VZ was determined for three cortical units of three different organoids. **(D)** Length of cilia at the apical side of the cortical units was measured using CiliaQ in three independent organoids and three cortical units per organoid.

All neurons described so far were generated through 2D differentiation protocols. Since an increasing number of studies are applying 3D protocols, which are closer to reproducing the complex tissue architecture, we also analyzed cilia in undirected cerebral organoids (Lancaster and Knoblich, 2014). As described with this protocol, we observed the formation of “cortical units” in 45-day-old cerebral organoids with proliferating progenitors organized around a lumen in a “ventricular zone” (VZ) and mature MAP2-positive neurons away from this lumen. The majority of ARL13B-positive cilia observed were found on the progenitor cells and were projecting into the lumen. In addition, we found more sparsely distributed ARL13B-positive cilia at the basal side of the cortical units on MAP2-positive neurons away from the VZ (Figure 7A). The ciliation rate of neuronal progenitors within the VZ was 61.81 ± 25.04% (n = 3 organoids) and the average cilium length was 1.465 ± 0.7189 µm (n = 272 cilia) (Figure 7B–7D), findings which are consistent with those described above for NSCs generated with 2D protocols. Given that cerebral organoids generated with this protocol contain a variety of cells outside of the VZ, which are challenging to comprehensively define with limited microscopy channels, and the difficulty in confidently assigning single cilia to MAP2+ cells, we refrained from trying to quantify ciliation rates outside of the VZ. This illustrates the challenges associated with analysis of these more complex 3D models.

### INPP5E and AC3 in neuronal primary cilia

**FIGURE 8:**
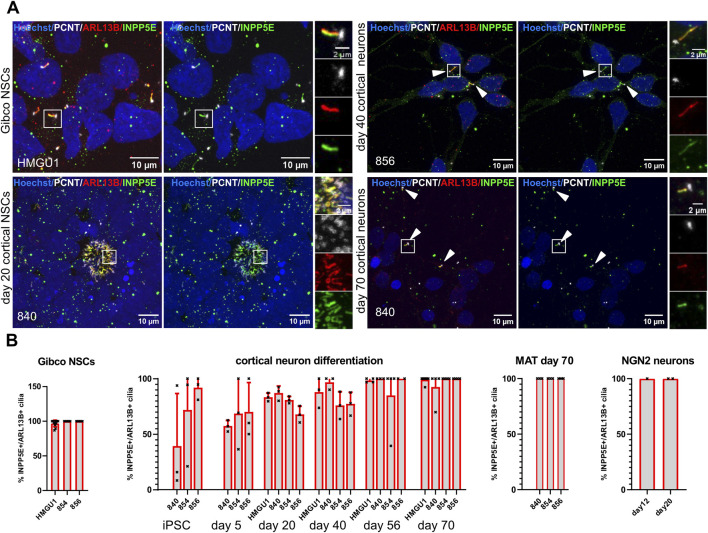
INPP5E is expressed in cilia of hiPSCs, NSCs and neurons. **(A)** Representative images (maximum projection of a confocal stack) of immunofluorescence stainings with anti-INPP5E and anti-ARL13B antibody in various neuronal cell types generated through different protocols as indicated. Each image is shown twice, with (left) and without (right) ARL13B staining, for better assessment of the INPP5E staining. Arrowheads point to sparse cilia. White boxes highlight the regions shown in the insets on the right with separate fluorescence channels. **(B)** Quantification of INPP5E+ cilia at all the differentiation stages and different protocols in the different lines: we assessed ciliation rate based on ARL13B-positive cilia (ARL13B+/PCNT+) and ciliation rate based on INPP5E-positive cilia (INPP5E+/PCNT+) separately and from these numbers calculated the percentage of INPP5E-positive cilia over ARL13B-positive cilia. Images of additional differentiation stages and protocols can be found in Supplementary Figures S16 and S17. Virtually all ARL13B+ cilia on neuronal cells are also INPP5E+. Note that we did not observe any cilia that were positive for INPP5E and PCNT but not ARL13B at any stage.

**FIGURE 9:**
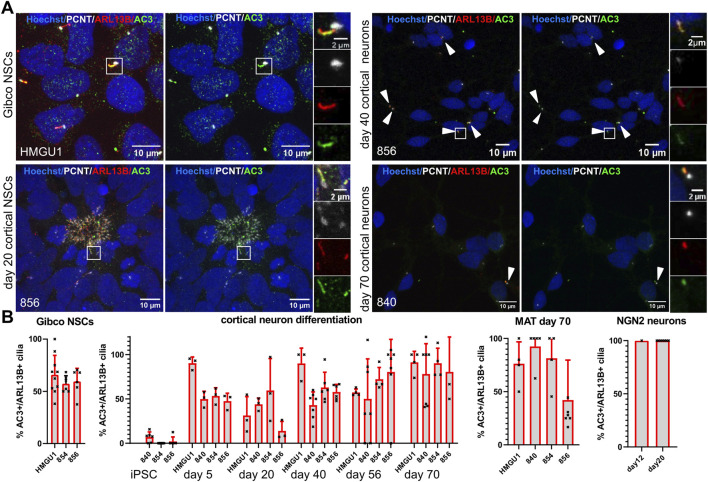
Adenylyl Cyclase 3 (AC3) is expressed on primary cilia of neuronal cells but not in iPSC cilia. **(A)** Representative images (maximum projection of a confocal stack) of immunofluorescence stainings with anti-AC3 and anti-ARL13B antibody in various neuronal cell types generated through different protocols as indicated. Basal bodies are marked with PCNT (white) and nuclei are counterstained with Hoechst. Each image is shown twice, with (left) and without (right) ARL13B staining, for better assessment of the AC3 staining. Arrowheads point to sparse cilia. White boxes highlight the regions shown in the insets on the right with separate fluorescence channels. **(B)** Quantification of AC3+ cilia at all the differentiation stages and different protocols in all the lines: we assessed ciliation rate based on ARL13B-positive cilia (ARL13B+/PCNT+) and ciliation rate based on AC3-positive cilia (AC3+/PCNT+) separately and from these numbers calculated the percentage of AC3-positive cilia over ARL13B-positive cilia. Images of additional differentiation stages and protocols can be found in Supplementary Figures S18 and S19. The proportion of AC3+ cilia increases with increasing maturity of the neurons.

Given that neurons *in vivo* are generally thought to be ciliated, we were intrigued by the reduction of ARL13B-positive cilia in the matured neurons. Since work in mouse suggested that neuronal cilia can be ARL13B-negative (Brewer et al., 2024), this raised the possibility that our analyses so far relying on ARL13B as a ciliary marker could have “missed” the presence of cilia. We were thus wondering if we can find cilia that are negative for ARL13B, but positive for other ciliary markers on these neurons. We systematically looked at expression of Inositol polyphosphate-5-phosphatase E (INPP5E) (Figure 8A) and Adenylate Cyclase3 (AC3) (Figure 9A) in combination with PCNT and ARL13B staining in the different neuronal differentiation protocols. We only occasionally observed apparently INPP5E-positive/ARL13B-negative or AC3-positive/ARL13B-negative cilia, but even in these cases, weak ARL13B signal overlapping with the INPP5E respectively AC3 signal could be detected upon further scrutiny, indicating that all cilia on neurons up to day 70 in culture analyzed here were at least weakly positive for ARL13B. Likewise, all cilia in GIBCO NSCs, day 20 cortical NSCs, day 40 cortical neurons and cerebral organoids, were positive for INPP5E, which was uniformly strong in all conditions (Figure 8B and Supplementary Figure S16). In day 45 cerebral organoids, INPP5E signal fully overlapped with ARL13B signal at the ventricular zone and on the cilia found on cells away from the VZ (Supplementary Figure S17). In contrast, AC3 signal was rarely seen on cilia in hiPSCs, but increased at day 5 of the cortical differentiation to about 50% positivity. On more mature day 70 NMM or MAT cortical neurons, as well as on NGN2 neurons, the majority of cilia were AC3 positive (Figure 9B and Supplementary Figure S18). In day 45 cerebral organoids we found AC3-positive as well as a few AC3- negative cilia, as defined by ARL13B-positivity, both within the ventricular lumen and at the basal side of the VZ (Supplementary Figure S19).

### GPR161 depletion from cilia upon SHH stimulation decreases in more mature neurons

**FIGURE 10:**
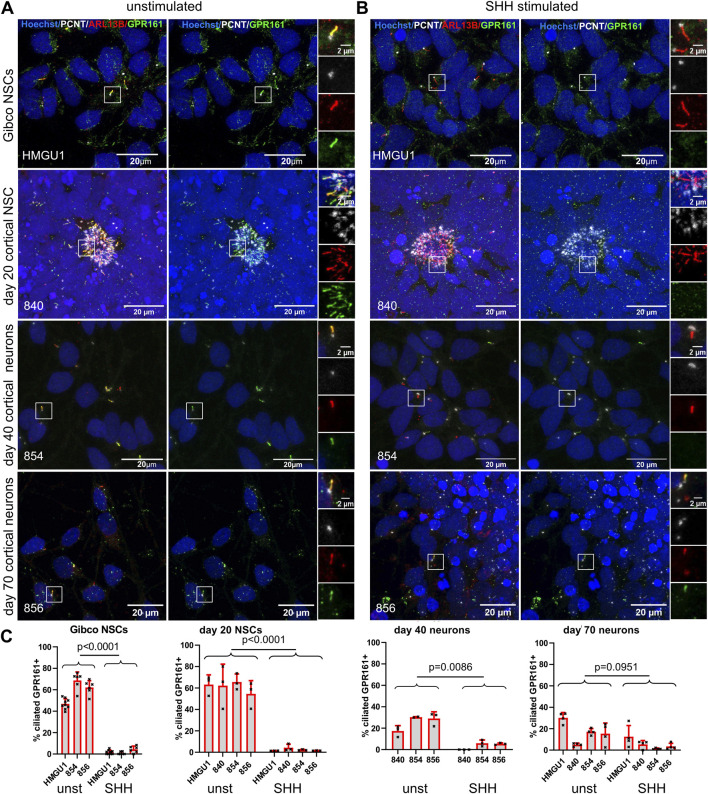
Ciliary response to SHH signaling as assayed by GPR161+ removal from cilia decreases in mature neurons. **(A and B)** Z-projected confocal microscopy images of Gibco NSCs, day 20 NCSs, day 40 and day 70 cortical neurons, stained with anti-ARL13B to mark cilia (red) and anti-PCNT to mark basal bodies (white), together with anti-GPR161 (green) to assess SHH signaling responsiveness. White boxes highlight the regions shown in the insets on the right with separate fluorescence channels. **(A)** Representative images of unstimulated cells (medium only was applied for 24 h). **(B)** Representative images of SHH-stimulated cells (medium supplemented with 100 ng/ml SHH was applied for 24 h). **(C)** Quantification of GPR161+ cilia (determined by the ratio of GPR161 signal over PCNT signal). Note the decrease in GPR161+ cilia after stimulation in NSCs and day 40 neurons but not in day 70 neurons. Statistical analysis was performed with an unpaired students t-test using the ciliation rates of individual FOVs. More GPR161 assays can be found in Supplementary Figure S20.

The G-protein-coupled receptor 161 (GPR161) is known to disappear from primary cilia upon SHH stimulation in the developing neuronal tube (Mukhopadhyay et al., 2013). We found GPR161 expressed in the majority of ARL13B-positive cilia in the Gibco NSCs, the dual SMAD inhibition cortical neuronal differentiations, the MAT and NGN2 neurons (Supplementary Figure S20). After 24 h of SHH stimulation, we observed a significant decrease of GPR161-positive cilia in the Gibco NSCs and in cortical differentiations at days 20 and 40 (Figure 10A–C). Importantly, the number of cilia, based on ARL13B staining, was not affected by SHH stimulation. These results indicate that cilia on NSCs and young neurons respond to SHH signaling as expected by removing GPR161 from their cilia. Interestingly, when we performed the same 24 h stimulation on more mature neurons (day 56 and 70 cortical neurons and MAT neurons), we observed a higher proportion of cilia with residual GPR161-positive cilia (Figures 10B,C and Supplementary Figure S20), suggesting a decrease in responsiveness to SHH pathway stimulation.

## Discussion

The key role of primary cilia in development and function of the human CNS remains only incompletely understood and requires further investigation. Human hiPSC-derived models now offer unprecedented opportunities to investigate the importance of ciliary biology in neuronal function and in disease of the CNS in humans. A growing number of laboratories worldwide therefore show interest in these methods and a plethora of different differentiation protocols to generate human neurons from hiPSCs are now available, such that the choice of the most appropriate protocol for a given scientific question becomes crucial. In light of this, we set out to perform a systematic analysis of primary cilia in various hiPSC-derived neuronal cell types using commonly used techniques based on immunofluorescence. Relevant findings include (1) the variable ciliation rate in hiPSCs across lines and batches, in opposition to the generally higher ciliation rate observed in neural stem cells (NSCs), (2) the decreasing ciliation rate with increasing neuronal maturity in 2D cell culture systems, strongly influenced by density of the cultures, (3) the differences in ciliary length and ciliation rate in NSCs generated from the same hiPSC line with different protocols and (4) the decreased responsiveness to hedgehog signaling of cilia in more mature neurons compared to NSCs and younger neurons.

Our findings bring a number of consequences for designing experiments. Given the importance of ciliopathies, which are Mendelian disorders caused by mutations in ciliary genes, many researchers aim at investigating the function of these genes by using patient-derived hiPSCs or by introducing mutations in these genes in control hiPSCs (Schembs et al., 2022; Mori et al., 2024; Wang et al., 2021; Wang et al., 2019). Furthermore, ciliary changes are now in focus beyond classical ciliopathies and assayed in models of neurodegenerative or metabolic diseases (Khan et al., 2021; Miyoshi et al., 2014; Fdez et al., 2022; Schmidt et al., 2022; Tian et al., 2023; Picon-Galindo et al., 2022). Typical assays involve measurement of ciliation rate and ciliary length to determine the role of the gene or condition of interest in ciliogenesis through comparison of mutant/diseased and control lines. Our findings underscore the importance of carefully controlled experiments if meaningful conclusions are to be drawn. On the one hand it is key to use multiple control lines (or closely related or isogenic lines) and on the other hand, the culture density must be taken into account and comparisons with respect to ciliation rates should only be made between areas of the culture with similar density. Moreover, a difference in ciliation rate may be biologically more relevant and robust at the NSC stage, where all protocols should yield more highly ciliated cultures, than at the hiPSC stage, where ciliation appears to be much more variable or at the stage of more mature neurons, where ciliation rates are very low in all lines and protocols.

Little data exists on quantifying ciliation rate in *in vivo* studies. The recent work by Wu et al. found a nearly 100% ciliation rate in neurons of all cortical layers in human tissue (Wu et al., 2024). Similarly, all neurons in murine primary visual cortex appear to be ciliated using a similar approach (Ott et al., 2024b). Likewise, murine hippocampal neurons *in vivo* appear to be largely ciliated. In other areas of the brain however, such as in mature cerebellar granule neurons, cilia are lacking, suggesting that not all mature neurons are ciliated (Ott et al., 2024a). In contrast to these *in vivo* studies, various *in vitro* experiments report lower ciliation rates. Only partial ciliation of cultured murine neurons has been described previously for choroid plexus with neuronal ciliation rates of 33% after 2–4 days in culture (Petralia et al., 2011) and hippocampal neuron ciliation rates of 60% after 7 days in culture (Berbari et al., 2007). Loss of primary cilia upon neuronal maturation in cultured cells has also been previously observed in human LUHMES cell-derived neurons (Coschiera et al., 2024). Hence, it appears that decreased ciliation is a common finding in 2D neuronal cultures.

While the surprising observation of low ciliation rates in neurons across all *in vitro* differentiation protocols used here is consistent with results from other *in vitro* studies, it raises a number of questions. While we cannot rule out that this represents a culture artefact, it may inform us on the role of cilia on neurons. Indeed, combined with the fact that ciliation rates are substantially higher with higher culture density, and in light of recent publications showing the “connectome” of neuronal cilia in a sample of human brain (Wu et al., 2024), this suggests an important role for cilia in communication with the cellular environment through direct physical interactions. This model is further supported by the recent description of active synapses on cilia (Sheu et al., 2022). By this rationale, in sparse cultures, neurons would disassemble their cilia due to lack of input. Primary cilia have so far been principally investigated during early development of the CNS, where their prominent role in transducing developmental signaling pathways, in particular hedgehog signaling, is well established. It is possible however, that cilia are no longer relevant in this context in the more mature brain and indeed, we do observe a decreased response to hedgehog pathway stimulation in the more mature neurons in culture. Hence, our findings are consistent with the emerging evidence that cilia on mature neurons play a very distinct role from that in differentiating cells, whereby they are involved in direct interactions with neighboring cells or their processes rather than in sensing diffusible molecules.

Several studies have analyzed primary cilia in hiPSC-derived neuronal models using immunofluorescence with one or more of the antibodies described here, albeit relying on other differentiation protocols and timing of analysis (Hembach et al., 2024; Mori et al., 2024; Schmidt et al., 2022). Overall, findings from these studies are however consistent with ours, showing high ciliation rates in NSCs and, when assessed, relatively low ciliation rates in neurons. Comparing ciliary length between these studies and ours is somewhat more challenging due to differences in the methodology (3D reconstruction of cilia versus measurement on 2D projections, inclusion of the basal body in the measurement or not, markers used, etc.). While 3D reconstructions are more cumbersome to perform, they probably provide a more reliable measure, especially given the ciliary orientation towards the medium in several of the protocols. Regardless of the methods employed, ciliary length in almost all of these studies was however substantially shorter than what has been recently described in the study by Wu et al. applying electron microscopy (Wu et al., 2024). Indeed, in that study, length of interneuron primary cilia was about ∼5 µm while cilia of projecting neurons reached 8 μm, in comparison to neuronal cilia in our studies falling below ∼2 µm in all protocols, except for the GIBCO NSCs which displayed cilia of up to 5 µm in length. These differences in ciliary length could be explained by cell-type specificity, but could also be influenced by various technical aspects such as fixation methods or duration.

Assessment of cilia is generally performed through immunofluorescence with antibodies against acetylated tubulin highlighting the axonemal microtubules and/or ARL13B, INPP5E or AC3 marking the ciliary membrane. Given that acetylated tubulin also marks all axons, this marker is not particularly useful in neurons due to very strong and abundant non-ciliary signal. By comparing the three membrane markers in various systems here, we confirm that INPP5E marks all cilia, from NSCs to neurons. In contrast to results from the mouse, ARL13B remains present in the cilia of the oldest neurons studied here after 70 days of differentiation, albeit with reduced intensity. Seeing the decreasing intensity, it may well be that these 70-day old neurons would completely lose ARL13B as well upon further maturation. Consistent with the mouse work, AC3 staining showed the opposite evolution during differentiation, being absent in hiPSCs and increasing with neuronal maturation. Our findings therefore support previous knowledge on expression of these ciliary markers and determine the best marker for each given differentiation stage.

Taken together, our results provide a systematic description of primary cilia in hiPSC-derived neuronal models, identifying crucial points to take into consideration when designing experiments to investigate the role of cilia in human neurons.

## Data Availability

The raw data supporting the conclusions of this article will be made available by the authors, without undue reservation.
