# Supplementary material for: Differences in neuronal ciliation rate and ciliary content revealed by systematic imaging-based analysis of hiPSC-derived models across protocols
Source: Front Cell Dev Biol. 2025 Apr 11;13:1516596. doi: 10.3389/fcell.2025.1516596 (PMC12021924; doi:10.3389/fcell.2025.1516596)
Supplement: Supplementary file 1 [file DataSheet1.pdf]

Supplementary Material

Differences in neuronal ciliation rate and ciliary content revealed by systematic imaging-based analysis of hiPSC-derived models across protocols

Walther Haenseler[1§], Melanie Eschment[2,3], Beth Evans[2], Marta Brasili[1,2], Joana Figueiro-Silva[4], Fee Roethlisberger[4,5], Affef Abidi[4,6], Darcie Jackson[4], Martin Müller[1,2], Sally A. Cowley[7], Ruxandra Bachmann-Gagescu[1,2,3,4§]

[1] URPP Adaptive Brain Circuits in Development and Learning, University of Zurich, Zurich, Switzerland

[2] Department of Molecular Life Sciences, University of Zurich, Zurich, Switzerland

[3] Clinical Research Priority Program Praeclare, University of Zurich, Zurich, Switzerland

[4] Institute of Medical Genetics, University of Zurich, Schlieren, Switzerland

[5] FHNW School of Life Sciences, University of Applied Sciences and Arts Northwestern Switzerland, Muttenz, Switzerland

[6] Institute of Regenerative Medicine, University of Zurich, Schlieren, Switzerland

[7] James and Lillian Martin Centre for Stem Cell Research, Sir William Dunn School of Pathology, University of Oxford, Oxford, UK

§ Corresponding authors:

Ruxandra Bachmann-Gagescu
Department of Molecular Life Sciences
Winterthurerstrasse 190
8057 Zurich
ruxandra.bachmann@mls.uzh.ch

Walther Haenseler
University Research Priority Program AdaBD
Winterthurerstrasse 190
8057 Zurich
Walther.haenseler@medgen.uzh.ch

Supplementary Figures

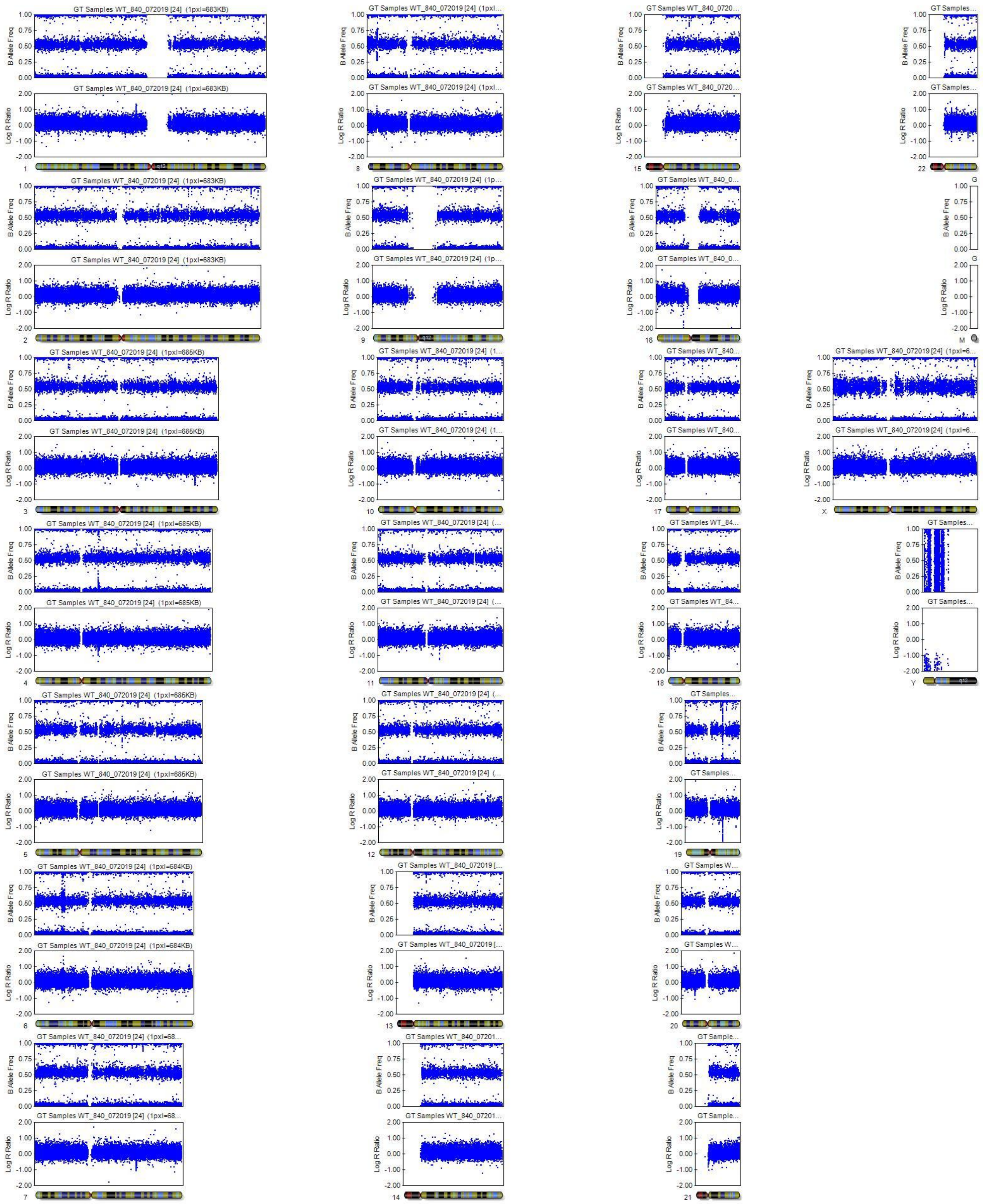

Figure S1: Quality control excluding major chromosomal aberrations in line SFC840-03-03: BeadChip GSA-24v3-0_A1. DNA isolated from hiPSC masterbatch passage 29, cultured in Essential 8 medium on Geltrex coated wells.

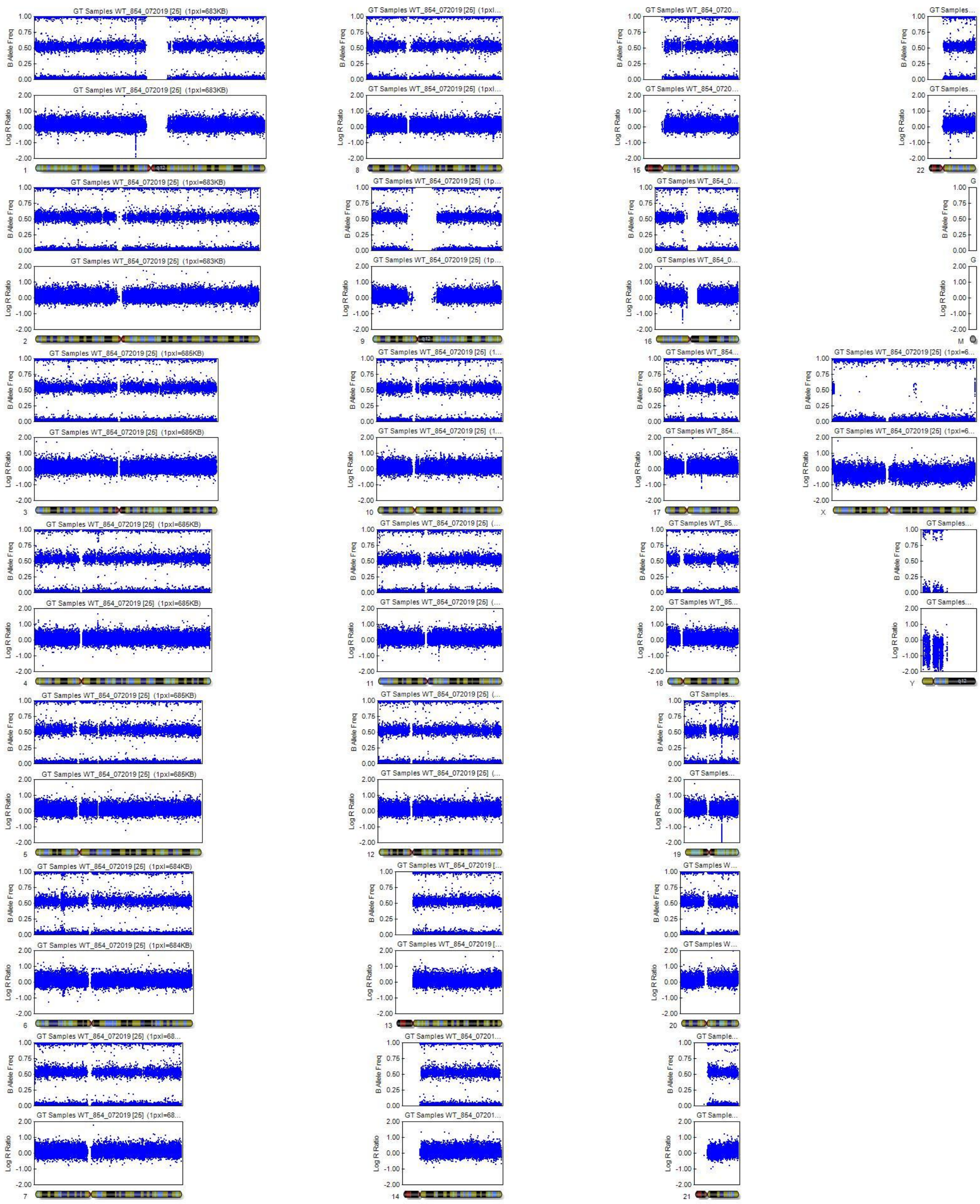

Figure S2: **Quality control excluding major chromosomal aberrations in line SFC854-03-02**: BeadChip GSA-24v3-0_A1. DNA isolated from hiPSC masterbatch passage 29, cultured in Essential 8 medium on Geltrex coated wells.

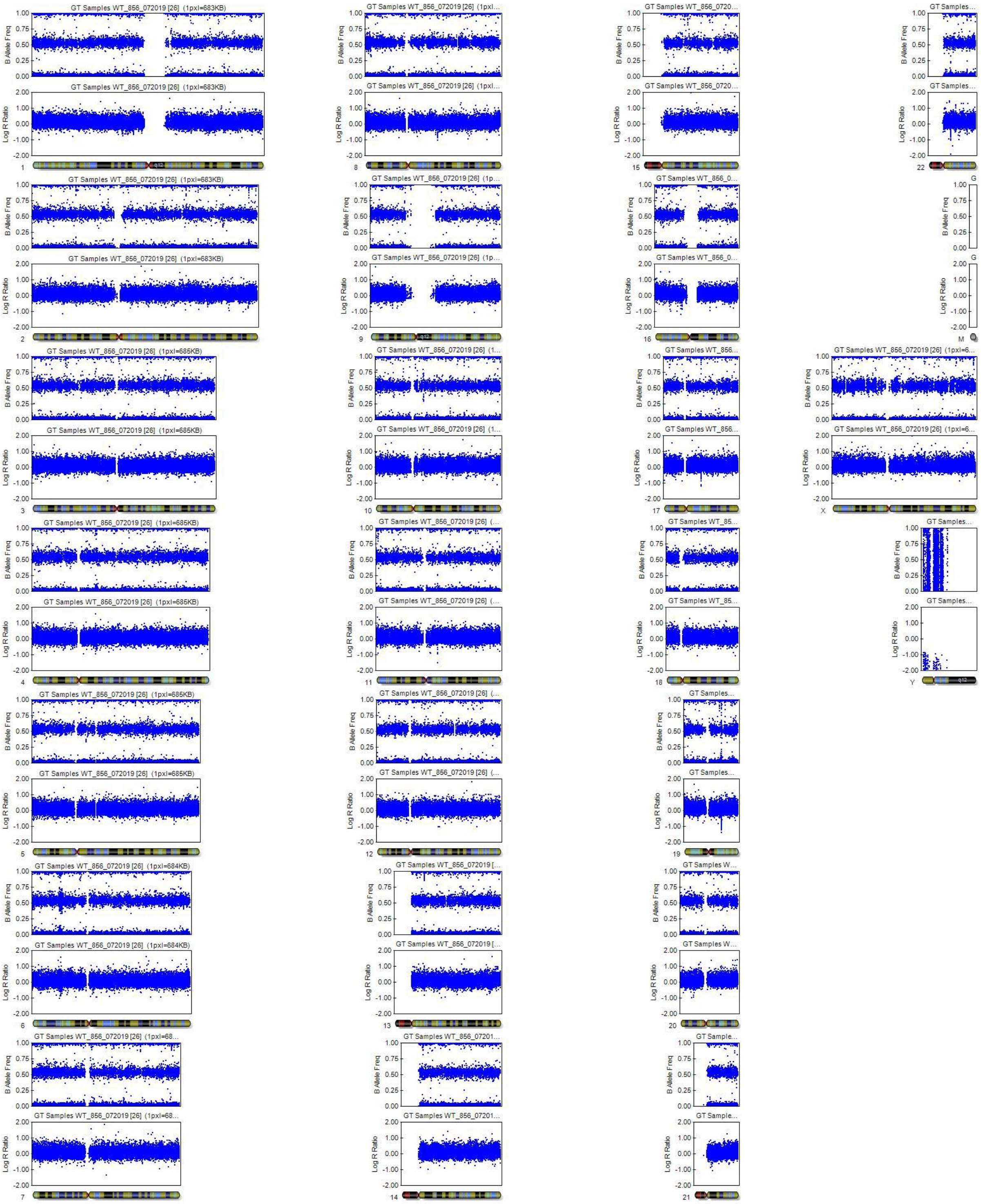

Figure S3: **Quality control excluding major chromosomal aberrations in line SFC856-03-04**: BeadChip GSA-24v3-0_A1. DNA isolated from hiPSC masterbatch passage 22, cultured in Essential 8 medium on Geltrex coated wells.

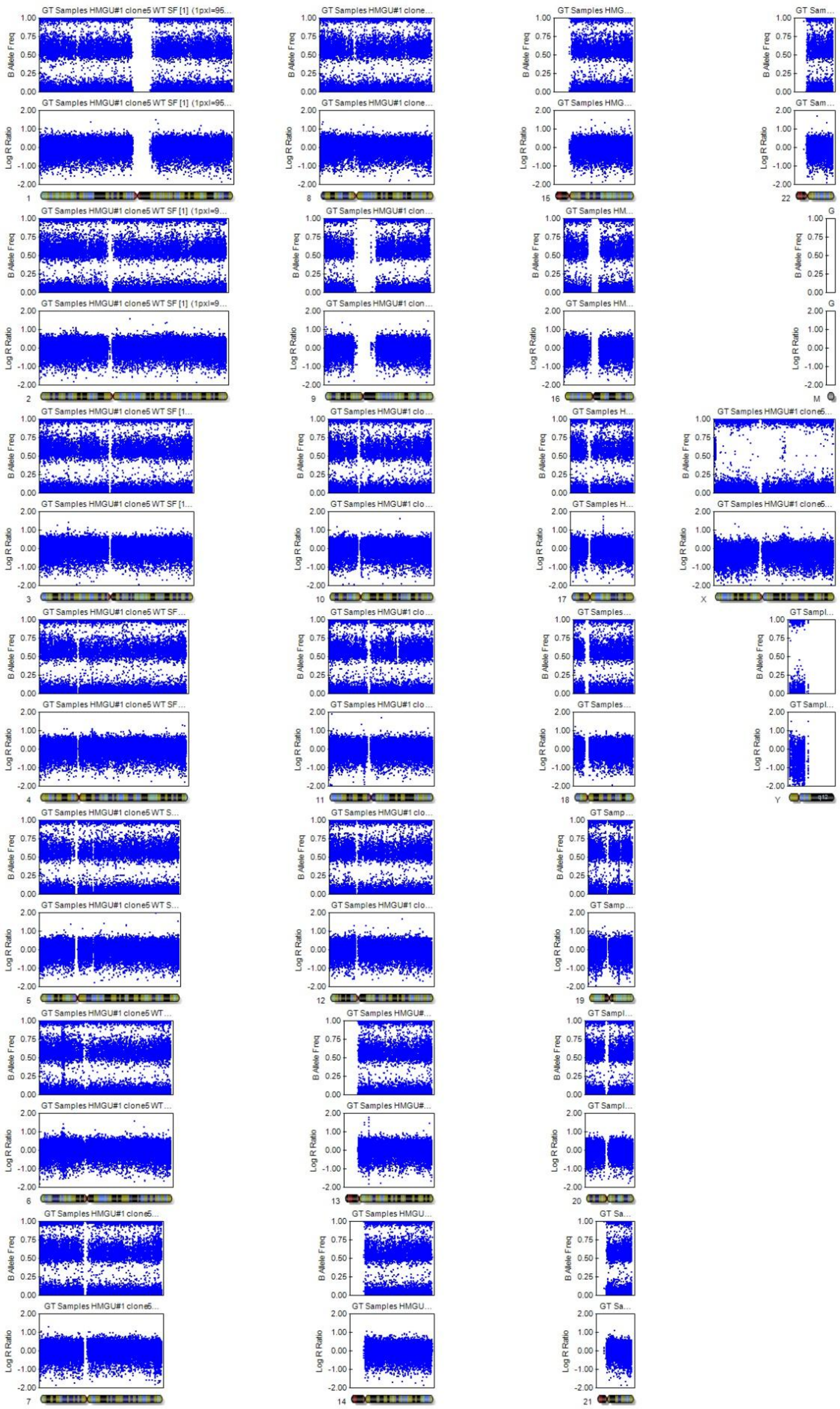

Figure S4: **Quality control excluding major chromosomal aberrations in line HMGU1 clone5**: BeadChip Infiniumhumanomniexpress-24v1-0_a. DNA isolated from hiPSC passage 28, cultured in StemFlex on Geltrex.

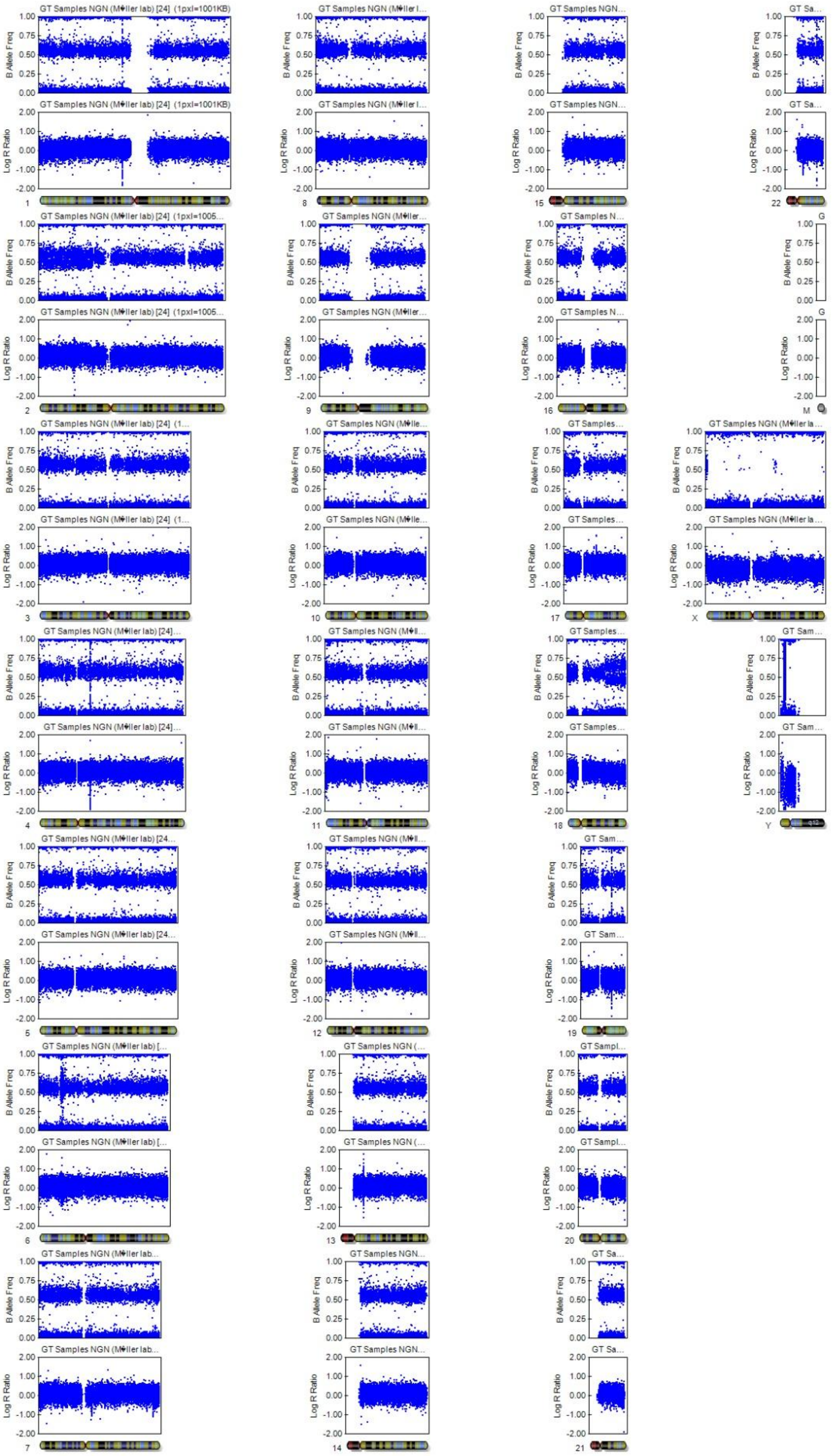

Figure S5: Quality control excluding major chromosomal aberrations in line WTC-11 i3N iPSC clone1: BeadChip Infiniumhumanomniexpress-24v1-0_a, cultured in Essential 8 medium on Geltrex coated wells.

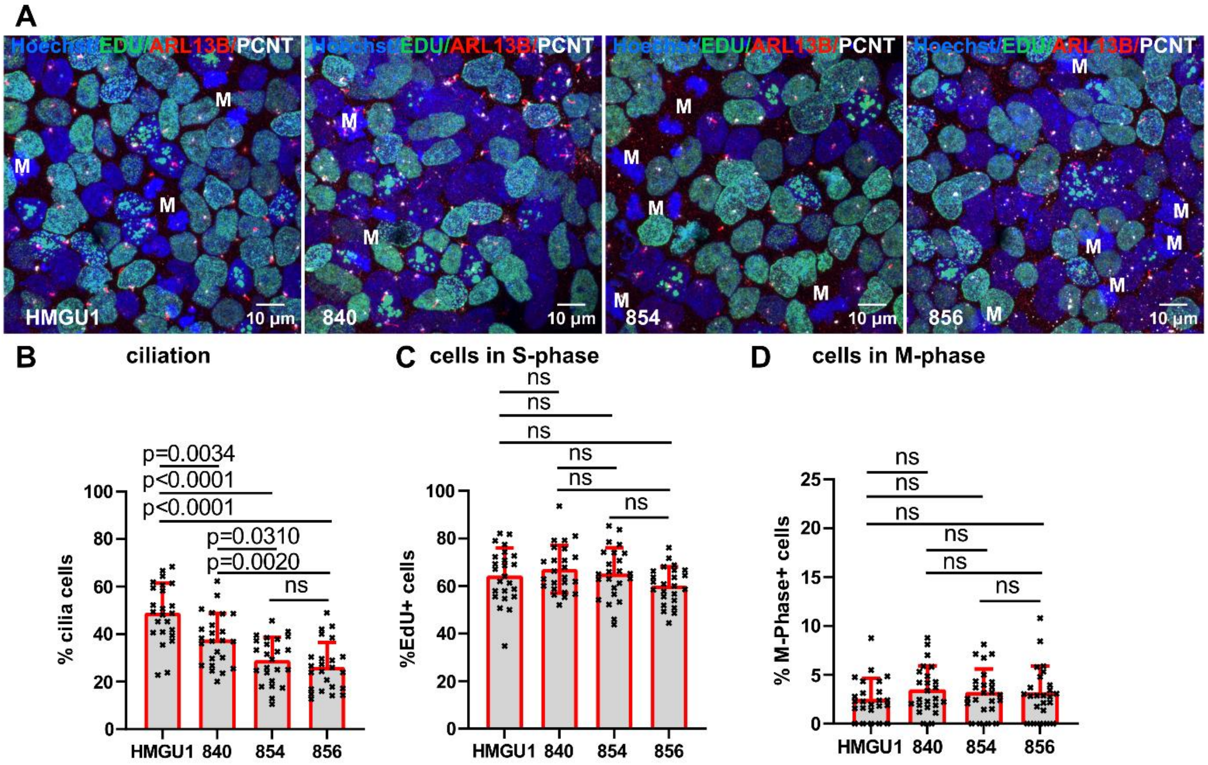

Figure S6: Ciliation rate is independent of proliferation rate in hiPSCs

Assessment of the proliferative state of the different hiPSC lines by EDU treatment (green) two days after plating before fixation, followed by immunostaining for PCNT (white) to mark basal bodies and ARL13B (red) to mark cilia. Images were acquired from 4 samples per line with 6 FOV per sample. **(A)** Representative images for each hiPSC line. **(B)** ciliation rate measured by ratio of ARL13B signal over PCNT signal. Note the wide range of ciliation rates within each line. **(C)** Cells in S-phase were detected with an EDU staining. %EDU+ cells were calculated by dividing number of EDU+ nuclei over the number of Hoechst+ nuclei. **(D)** Cells in M-phase were identified with the HOECHST staining as cells with condensed chromosomes (M). %M-phase cells+ cells were calculated by dividing number of M-phase+ nuclei over the total number of Hoechst+ nuclei. Statistical analysis was performed with a Tukey’s multiple comparison test.

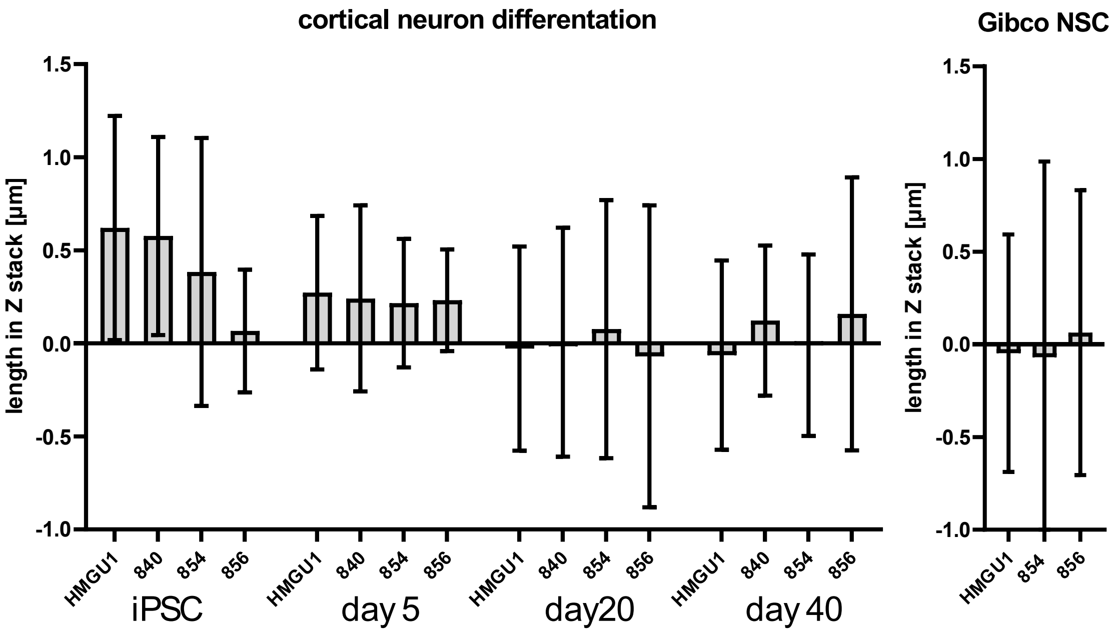

Figure S7: Orientation of primary cilia in the z-plane in hiPSCs and in 2D neuronal differentiations

Cilia orientation of cilia was calculated based on x,y,z coordinates provided by CiliaQ. The length of cilia in the z-direction is plotted as a bar graph showing the mean +/- SD for each hiPSC line at different timepoints during the cortical neuronal differentiation protocol (Shi et al) and Gibco NSCs. A positive value means the cilia face towards the medium, a negative value that they face towards the plastic of the tissue culture dish. Note the positive orientation for hiPSC and NSCs at day 5 of differentiation in the cortical neuron protocol. At other timepoints and with the Gibco protocol, the orientation in the z-plane was more random.

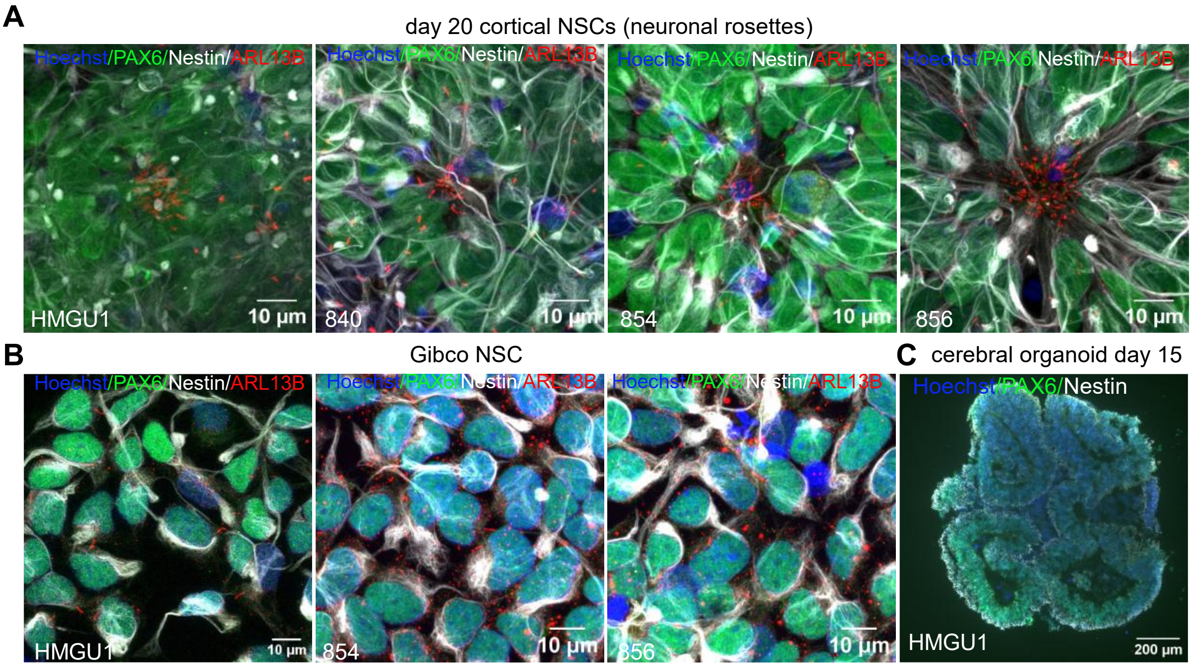

Figure S8: hiPSC-derived NSCs express typical markers PAX6 and Nestin

Quality control for hiPSC-derived NSCs using immunostainings with anti-PAX6 antibody (green) and anti-Nestin antibody (white). Cilia are marked with anti-ARL13B (red). **(A)** Most cells in day 20 neuronal rosettes generated with the dual SMAD inhibition differentiation protocol express the NSC markers PAX6 and Nestin. Representative maximum projection images of z-stacks for all differentiated hiPSC lines are shown. Note the polarization of NSCs into rosettes with cilia in the apical part of the cell pointing into a “lumen”. **(B)** The majority of NSCs generated using the Gibco protocol are positive for the same NSC markers PAX6 and Nestin but note that these cells are not polarized. **(C)** Day 15 cerebral organoids are composed of NSCs expressing PAX6 and Nestin.

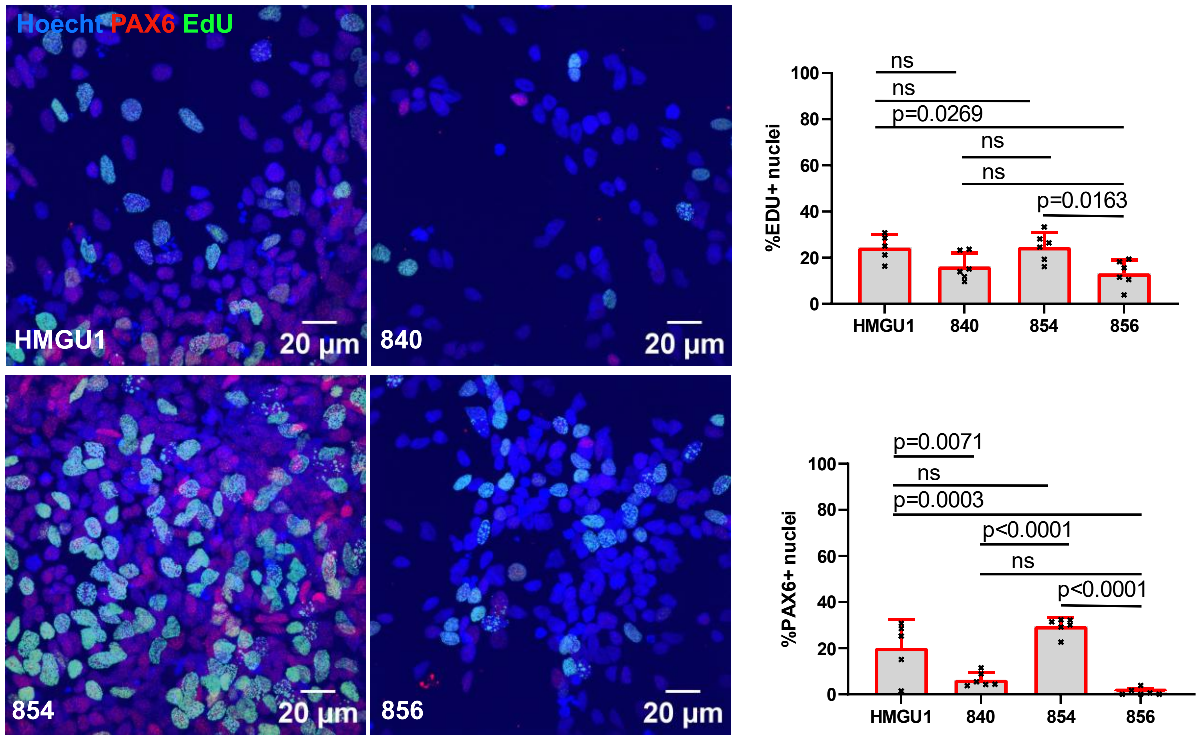

Figure S9: Assessment of proliferating progenitor cells in day 40 cortical neurons

Representative Z-projected confocal images of immunostained dual SMAD inhibition cortical neurons at day 40 with HOECHST (blue) to stain all nuclei, EdU (green) to stain the cells in S-phase and PAX6 (red) to identify remaining neuronal stem and progenitor cells. Quantifications are made from 6 FOV per iPSC line. Nuclei were reconstructed with IMARIS and the mean fluorescence intensity of EDU, respectively PAX6 in the reconstructed nuclei was calculated. A fixed intensity threshold was applied to all images to define EDU+ and "strongly" PAX6+ nuclei and they were divided by the total number of nuclei to obtain % EDU+ nuclei respectively % strongly PAX6+ nuclei. Statistical analysis was performed with a Tukey's multiple comparison test.

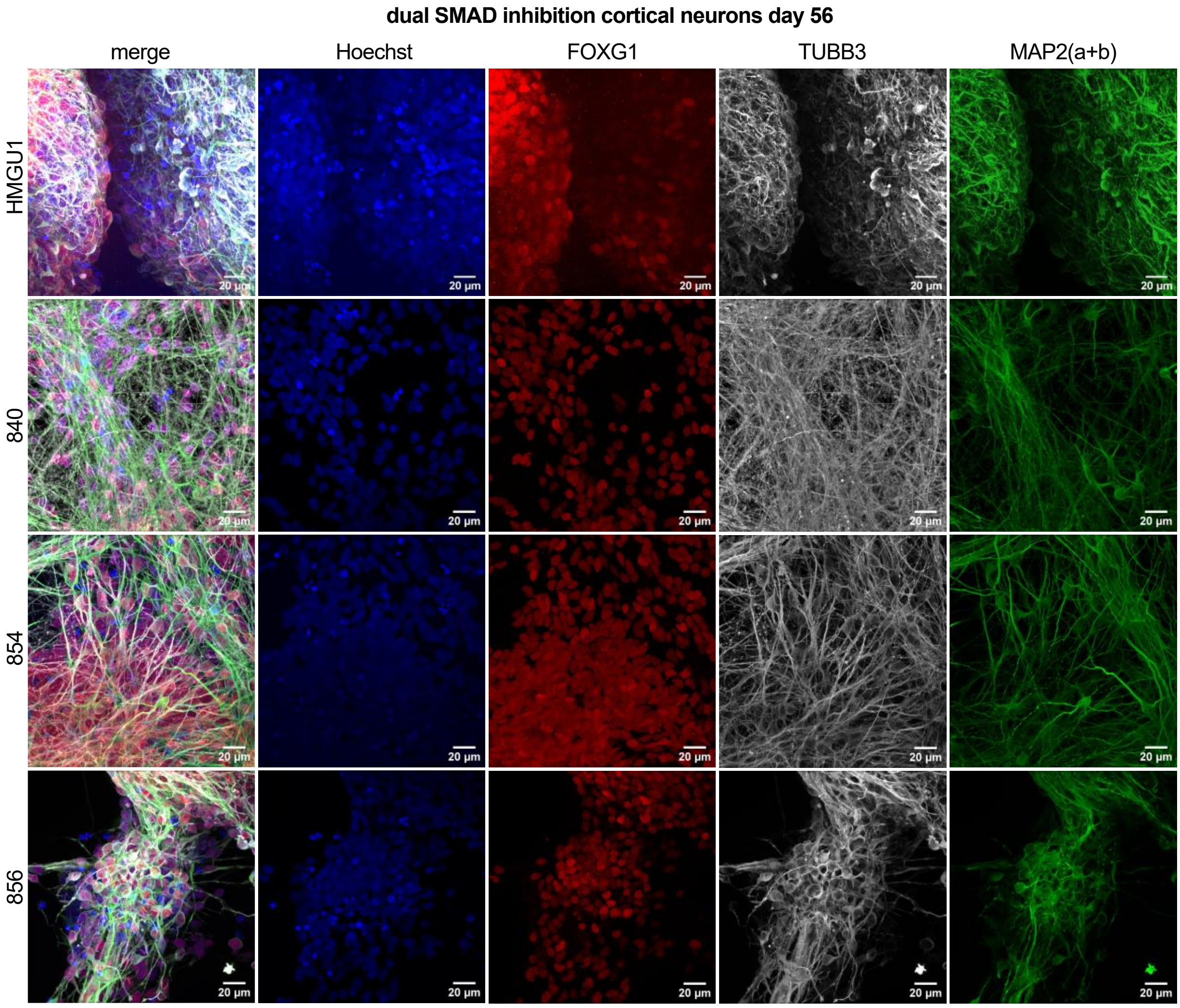

Figure S10: Cortical neurons produced with the dual SMAD inhibition protocol display forebrain commitment

Z-projected confocal images of immunostained neurons at day 56 with antibodies against MAP2(a+b) (green) marking mature neurons and their processes, TUBB3 (white) highlighting neurites and forebrain commitment marker (FOXG1 -red). Nuclei are counterstained with Hoechst. Note that most cells are positive for the forebrain commitment marker FOXG1 at day 56.

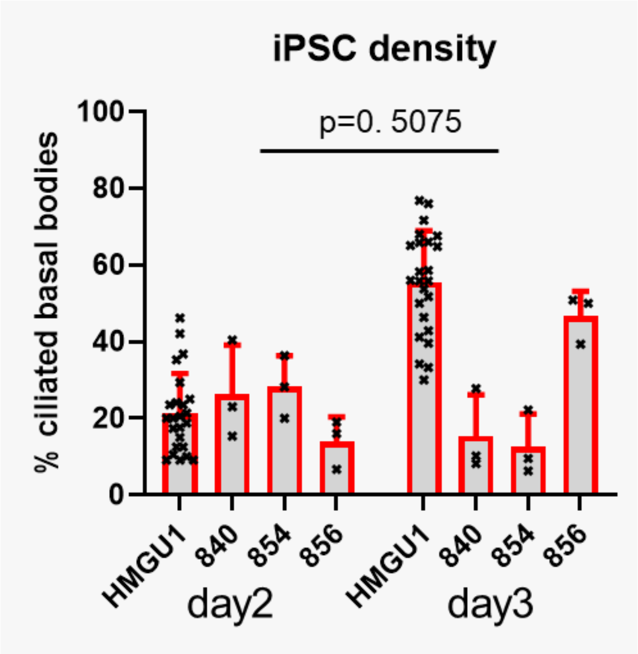

Figure S11: Effect of hiPSC culture density on ciliation rate

Assessment of ciliation rate of hiPSCs at day 2 after EDTA passage (where the culture consists of separate hiPSC colonies with a confluency of around 50%) and at day 3 (paired samples) after the same passage (when hiPSCs are packed more densely with a confluency over 90%). Crosses represent individual fields of view. Statistical analysis was performed with a paired t-test of the mean ciliation rate for each hiPSC line. Red bar plots show mean +SD.

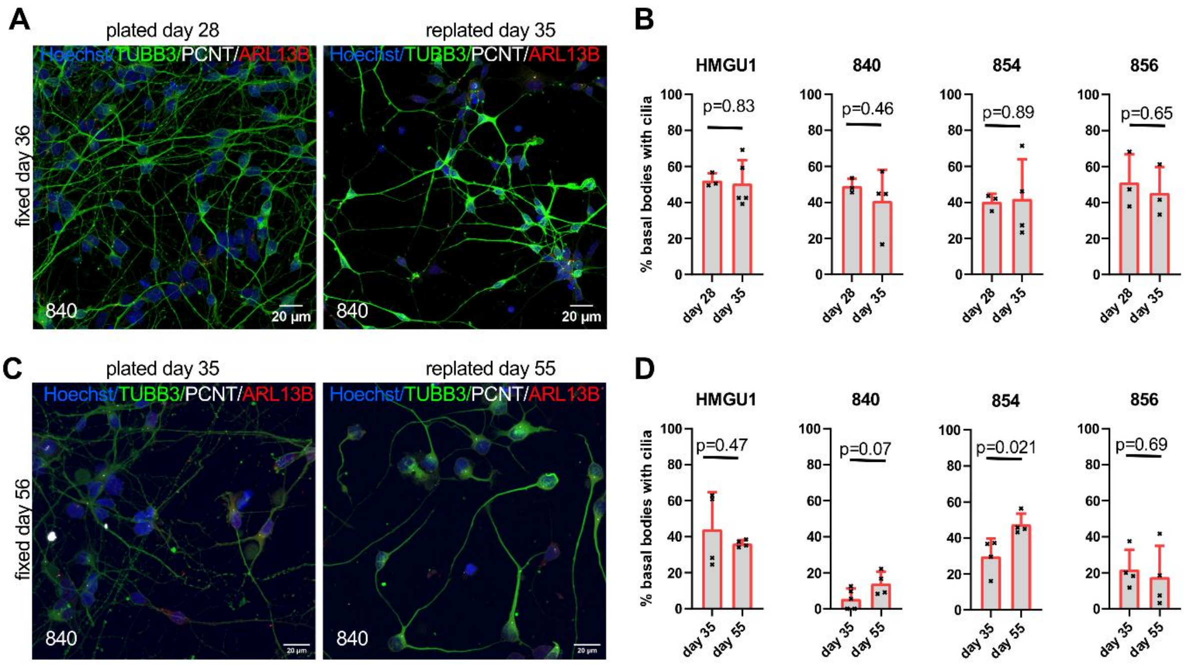

Figure S12: Effect of replating on ciliation rate.

According to the protocol by Shi et al, standard final plating of cortical neurons is at day 35. **(A)** We compared standard final plating into the imaging plate at day 35 (right panel) to thawing neurons directly into the imaging plate at day 28 (left panel). In both cases, cells were fixed at the same timepoint at day 36. Images are Z-projected confocal images of immunostainings highlighting neurons with their neurites using anti-TUBB3 antibody (green), cilia with anti-ARL13B (red) and basal bodies with anti-pericentrin (PCNT, white). **(B)** Quantification of ciliation rate (expressed as % ARL13B+ cilia over PCNT+ basal bodies) in each of the four lines without replating ("day28") or with replating at day 35 ("day 35"). No statistically significant difference was observed with replating. **(C)** We compared standard final plating into the imaging plate at day 35 to replating neurons again into the imaging plate at day 55, with fixation at day 56 in both cases. Same stainings as in **(A)**. **(D)** Same quantification as in **(B)**, comparing this time cells replated once ("day 35") and those replated again at day 55 ("day 55"). Again, no influence of late replating was observed. Individual datapoints represent one field of view, bar graphs show the mean and SD. Statistical analysis was performed with an unpaired students t-test using fields of view.

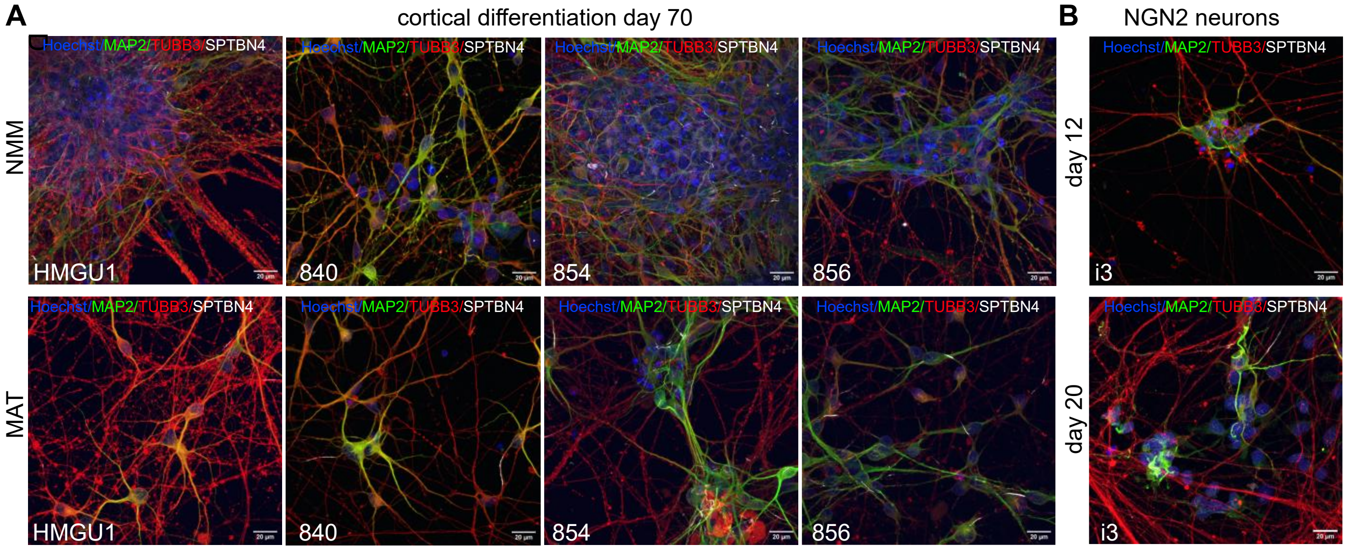

Figure S13: Comparison of cortical neuronal maturation in different differentiation protocols

Z-projected confocal images of immunostainings highlighting mature neurons (MAP2 – green), neurites (TUBB3 - red) and axon initial segments (beta-spectrin SPTBN4 – white). **(A)** Neurons at day 70 were either matured in neural maintenance medium (NMM) or in maturation (MAT) medium. Neuronal cultures generated in MAT medium exhibit lower but less variable neuronal density than those generated in NMM medium, with increased amounts of MAP2+ neurons and presence of SPTBN4+ axon initial segments in MAT neurons. **(B)** Most of the NGN2 induced neurons were positive for TUBB3 and MAP2 at day 12 and day 20. SPTBN4+ axon initial segments were present at day 20 but not at day 12 after NGN2 induction. Scale bars are 20 µm in all panels.

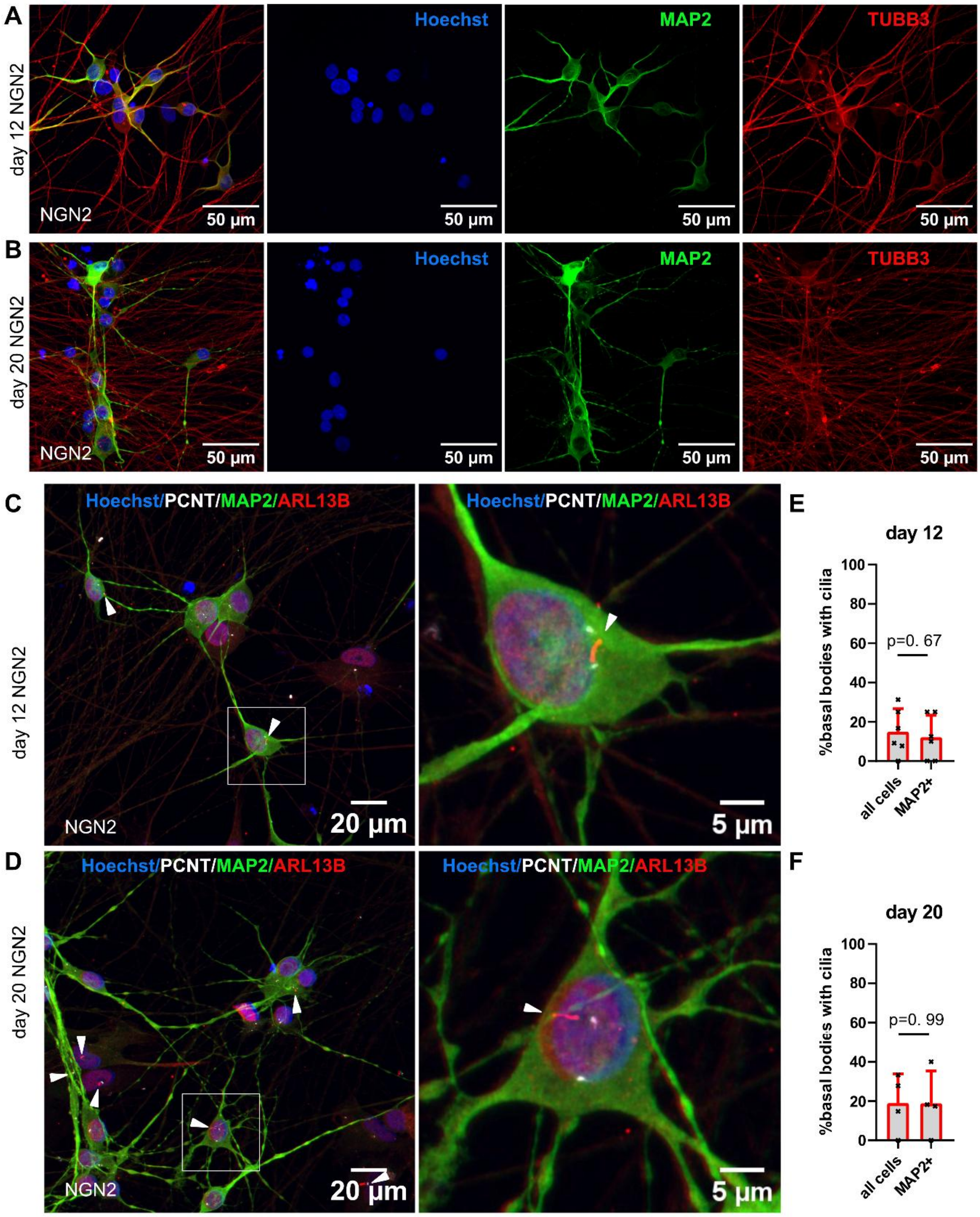

Figure S14: Analysis of cilia restricted to mature MAP2+ cells in neuronal cultures generated with the NGN2 protocol

(A-B) Z-projected confocal images of immunostainings highlighting mature neurons (MAP2 – green) and neurites (TUBB3 - red) on day 12 **(A)** and day 20 **(B)** after NGN2 induction. Note that there are also a few MAP2- cells in the images. **(C-D)** Z-projected confocal images of immunostainings highlighting mature neurons (MAP2 – green) and cilia (ARL13B – red) with basal bodies (pericentrin PCNT – white) on day 12 **(C)** and day 20 **(D)** after NGN2 induction.

The left panels show the overview image where the white box indicates the zoomed in region shown in the right panels. White arrowheads indicate cilia. **(E, F)** Ciliation rate shown for all cells (all nuclei), respectively restricted to cilia found on MAP2+ neurons. Crosses represent fields of view and red bar plots show mean + SD. Statistical analysis was performed with an unpaired t-test using fields of view at day 12 and day 20.

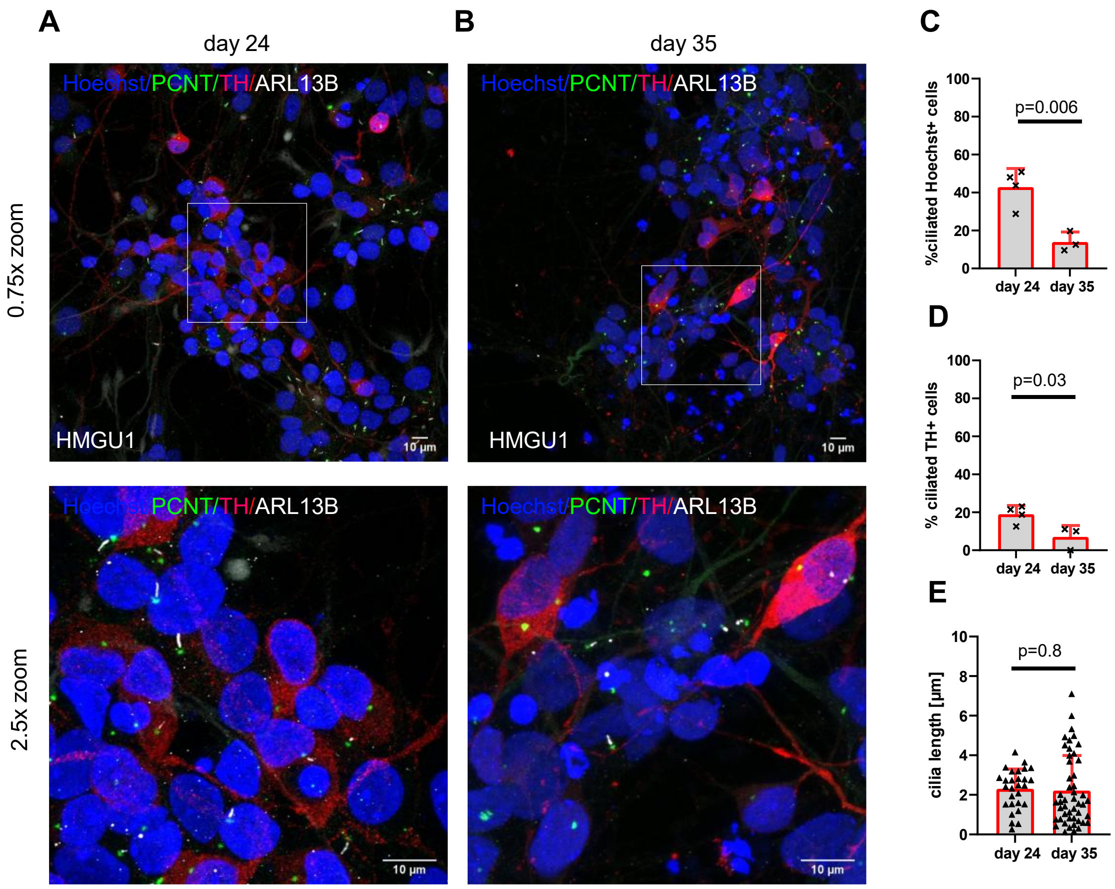

Figure S15: Cilia on dopaminergic neurons

(A-B) Z-projected confocal images of immunostainings highlighting dopaminergic neurons (tyrosine hydroxylase TH positive – red) with cilia (ARL13B – white) and basal bodies (pericentrin PCNT – green) at day 24 **(A)** and day 35 **(B)** of differentiation. Nuclei are counterstained with Hoechst. Overview images on top with zoomed in images on bottom (boxed region shown in overview images). **(C)** Quantification of ciliation rate in % expressed as number of ARL13B/PCNT positive cilia over number of Hoechst positive nuclei. **(D)** Quantification of ciliation rate in % expressed as number of ARL13B/PCNT positive cilia over number of TH positive cells. Note that the ratio of ciliated cells on TH+ neurons decreases with increasing culture time. **(E)** Cilia length was quantified at day 24 and day 35 of differentiation in the 2.5x zoom images using CiliaQ. No significant difference in average length of cilia was observed between the two timepoints. (C-D) Each datapoint represents the mean of multiple fields of view (FOVs) at 0.75x zoom from an independent neuron induction run of the HMGU1 hiPSC line. (E) Each datapoint represents a single cilium. Statistical analysis was performed with an unpaired t-test.

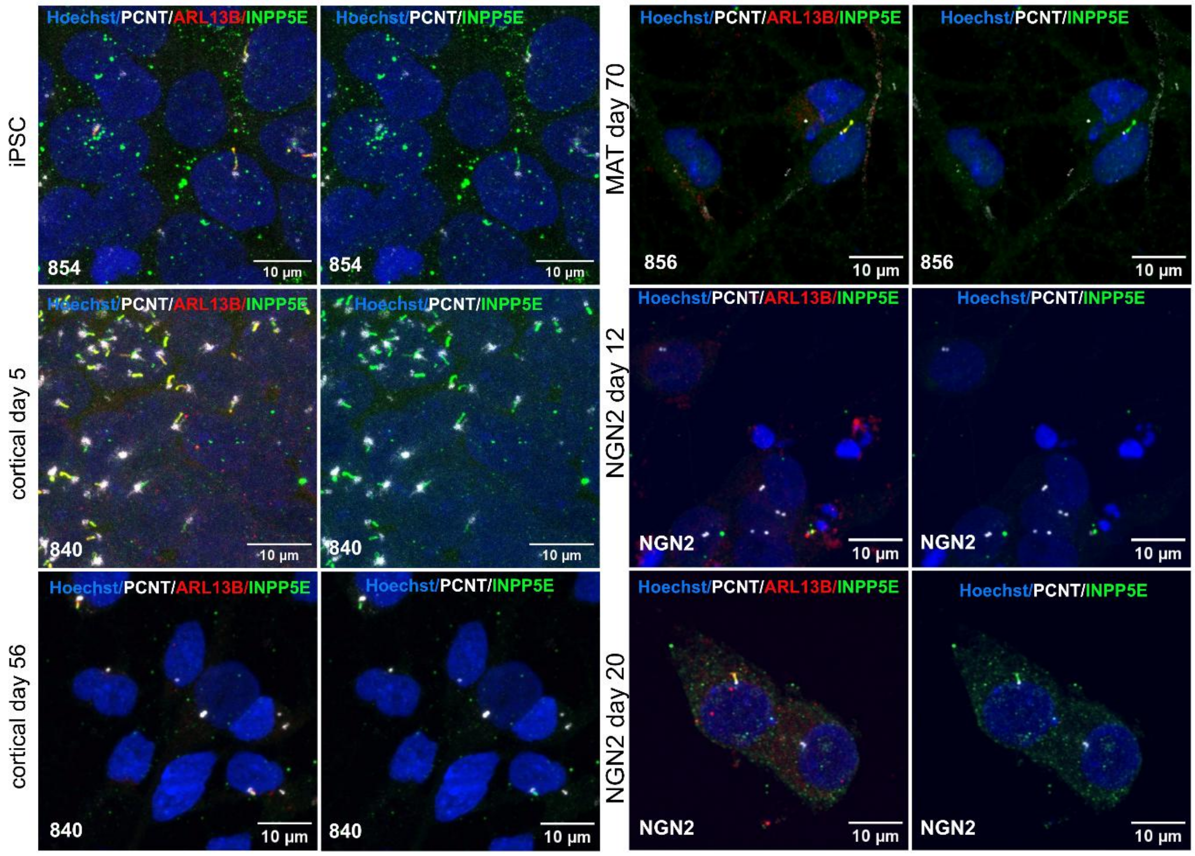

Figure S16: INPP5E additional stainings

Representative images (maximum projections of confocal stacks) of immunofluorescence stainings with anti-INPP5E and anti-ARLB13B antibody in various neuronal cell types generated through different protocols as indicated. Each image is shown twice, with (left) and without (right) ARL13B staining, for better assessment of the INPP5E staining.

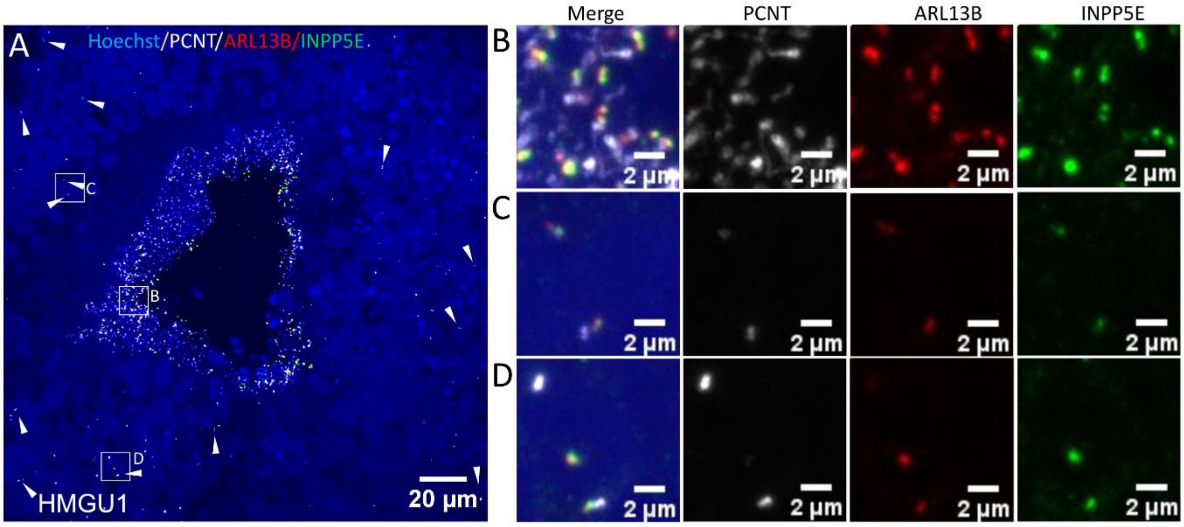

Figure S17: Cilia of cerebral organoids are INPP5E+

Z-projected confocal image of a 45-day-old cryosectioned cerebral organoid stained for ciliary membrane markers anti-ARL13B (red) and anti-INPP5E (green). Basal bodies are marked with anti-pericentrin (PCNT, white) and nuclei are counterstained with Hoechst. **(A)** Overview image showing a cortical unit with the lumen in the middle bordered by progenitors. Boxes indicate the zoomed in images **(B-D).** Note that most cilia line the “ventricular zone” (VZ); arrowheads point to isolated cilia outside of this VZ. All ARL13B+ cilia are also INPP5E+.

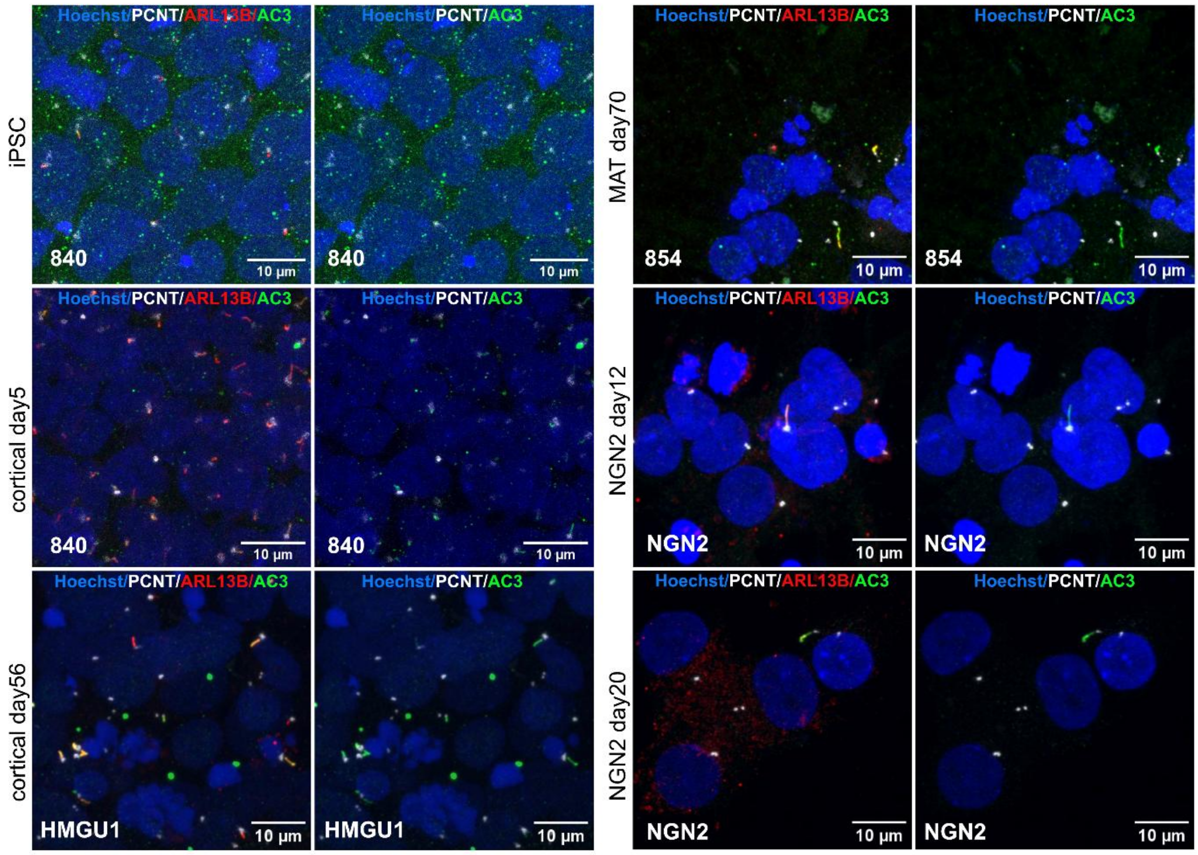

Figure S18: AC3 additional stainings

Representative images (maximum projection of a confocal stack) of immunofluorescence stainings with anti-AC3 and anti-ARLB13B antibody in various neuronal cell types generated through different protocols as indicated. Each image is shown twice, with (left) and without (right) ARL13B staining, for better assessment of the AC3 staining.

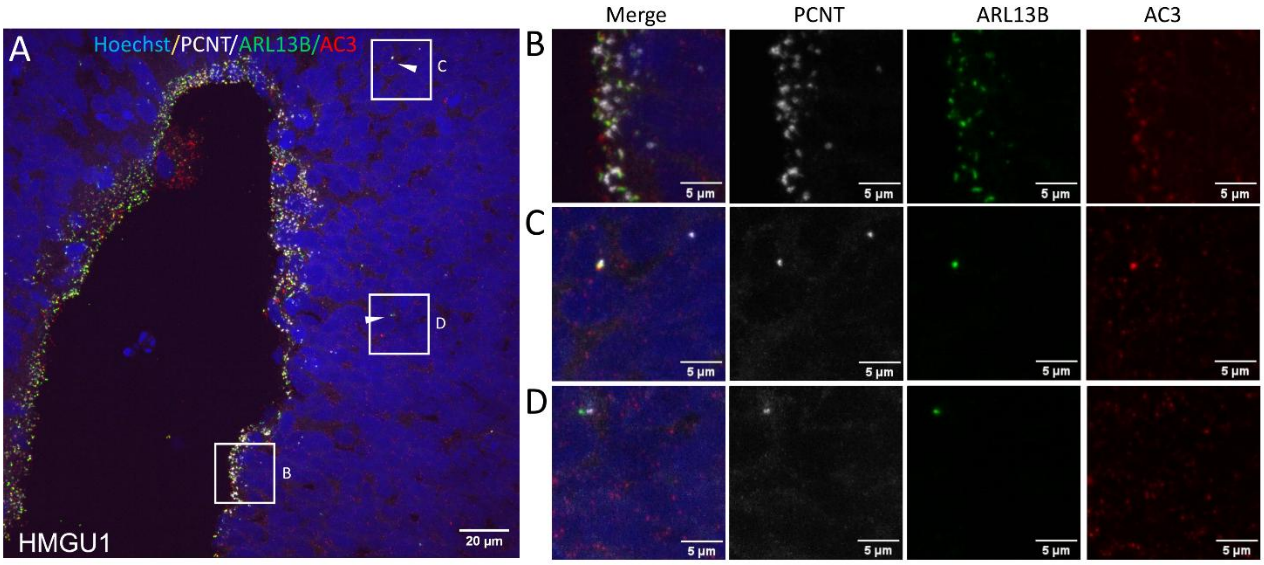

Figure S19: Cilia of cerebral organoids are partially AC3+

Z-projected confocal image of a 45-day-old cryosectioned cerebral organoid stained for ciliary membrane markers anti-ARL13B (green) and anti-adenylyl cyclase 3 (AC3, red). Basal bodies are marked with anti-pericentrin (PCNT, white) and nuclei are counterstained with Hoechst. **(A)** Overview image showing a cortical unit with the lumen in the middle bordered by progenitors. Boxes indicate the zoomed in images **(B-D).** Note that most cilia line the ventricular zone. The majority of ARL13B+ cilia are also AC3+, but **(D)** gives an example of an AC3- cilium.

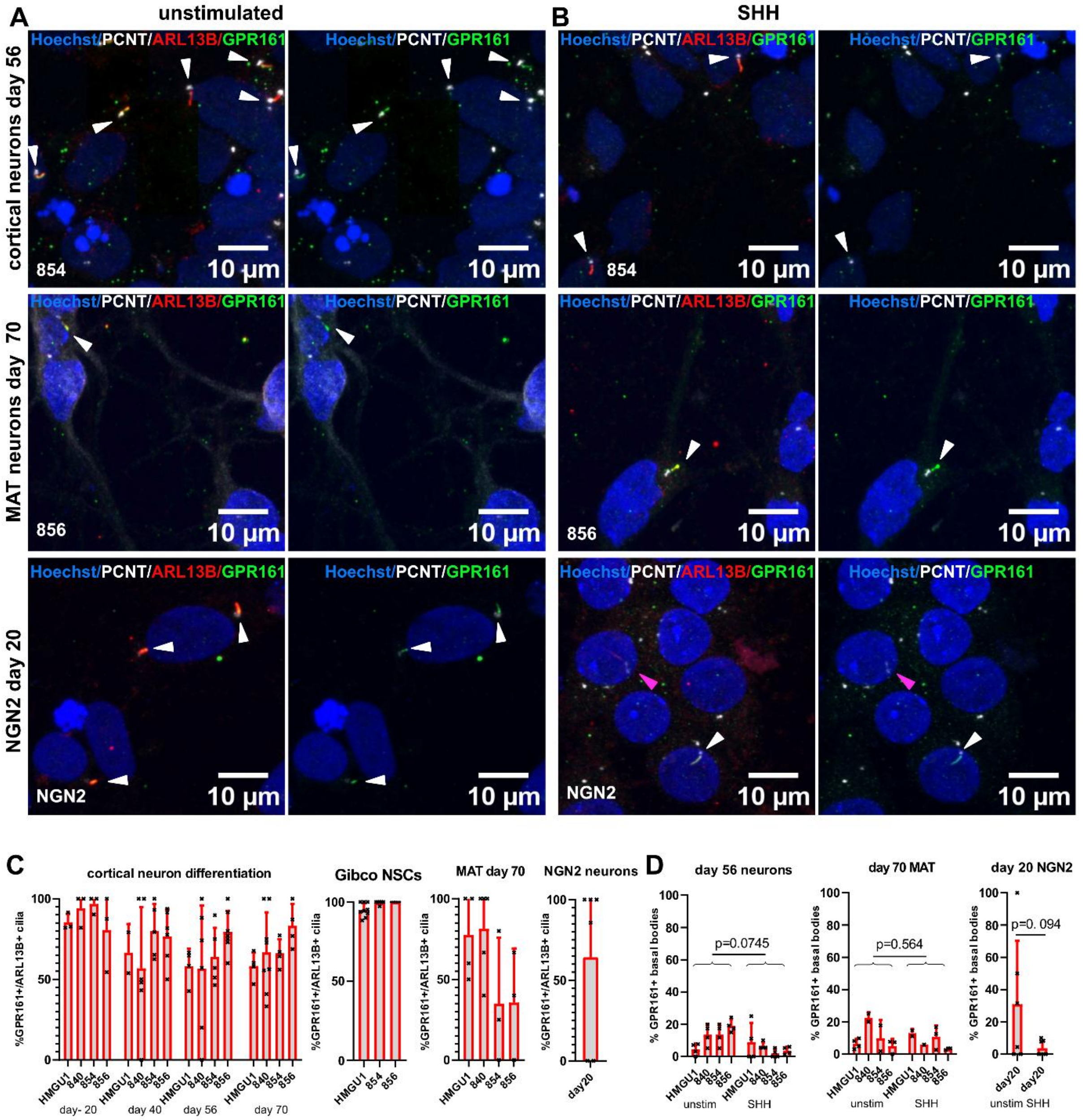

Figure S20: Hedgehog stimulation additional assays

(A-B) Representative maximum projection confocal images of unstimulated (left) and SHH stimulated (right) neurons. White arrowheads indicate GPR161+ cilia, whereas pink arrowheads point to GPR161 negative cilia. **(A)** Representative images of unstimulated cells (medium only was applied for 24h). Note that all cilia are GPR161+ as expected in the absence of SHH ligand. **(B)** Representative images of SHH stimulated cells (medium with 100 ng/ml SHH was applied for 24 h). Hedgehog stimulation should lead to removal of GPR161 from the cilium; note that the majority of cilia in these neurons continue to be GRP161+. **(C)** Quantification of GPR161+ cilia expressed as the ratio of GPR161+ over ARL13B+ cilia. **(D)** Quantification of GPR161+ cilia over PCNT+ basal bodies with (left side of the graph) and without (right side of the graph) SHH stimulation. Note that neurons at day 56 or 70 show no

significant downregulation of ciliary GPR161 upon SHH stimulation. For NGN2 neurons at day 20, some cilia lose GPR161 (pink arrowhead), while others do not respond to SHH stimulation maintaining GPR161 (white arrowhead). Each datapoint represents one field of view, bars indicate mean + SD. Statistical analysis was performed with an unpaired students t-test using the ciliation rates of individual fields of view.

Supplementary Methods

1. Cortical differentiation

Cortical differentiation was performed following a previously described dual SMAD inhibition protocol (Shi et al. 2012), with minor modifications:

hiPSCs cultured on Geltrex in Essential 8 medium (E8) were passaged 2 days before induction as small cell clusters using 0.5 mM EDTA into a well of a Geltrex-coated 6 well plate. On the day of induction, the iPSCs needed to reach a confluency of more than 90%. E8 was removed, cells were washed with PBS and 5 ml of neuronal induction medium (NIM) was applied. For the next 12 days 5 ml of NIM was replaced daily. On day 12 medium was first replaced with 2 ml NIM, then 200 µl of dispase2 (10 mg/ml in PBS, freshly sterile filtered) was added to the medium and the neuroepithel was cut in 16 sheets with the pipette tip. The cells were incubated until most of the sheets had lifted (around 10 minutes), then the sheets were carefully transferred to a tube containing 10 ml warm advanced DMEM/F12. We waited for gravity settling of the sheets, which took about 1 minute, then medium was removed and 10 ml fresh advanced DMEM/F12 was added for an additional wash by gravity settling. After 3 washes the sheets were carefully taken up in 4 ml NIM and transferred to 2 wells of a Geltrex coated 6 well plate. The next day the medium was replaced with 3 ml neuronal maintenance medium (NMM) + 20 ng/ml bFGF and 2 days later the medium was again replaced with 3 ml NMM +20 ng/ml bFGF. From day 17 onwards the neurons were only cultured in NMM with medium changes every 2-3 days. By day 18 neuronal rosettes formed by the neuronal stem cells (NSCs) were visible and dispase2 was used to lift the rosettes formed by NSCs and to transfer them to a new dish without breaking up the neuronal rosette structure. For this we fed the neurons with 2 ml fresh NMM and applied 200 µl dispase2 to the medium. After about 10 minutes the neuronal rosettes had lifted, whereas other cells remained attached to the cell culture dish. The lifted rosettes of 2 wells of a 6 well plate were carefully collected and transferred to a tube with 10 ml advanced DMEM/F12 and washed 3 times by gravity settling as described before. Depending on the amount of collected material, the rosettes were now plated to 1 or 2 wells of a Geltrex coated 6 well plate in 2 ml NMM for further culture, respectively for imaging on polyornithine/laminin coated 96 well Ibidi plates. NMM was replaced the following day. On day 22 neuronal rosettes were again passaged with dispase2 to a new Geltrex coated 6 well plate. By day 28 we obtained a mix of NSCs organized in rosettes and of cells with neuronal morphology. Here we froze the cells in NMM +10% DMSO for cryostorage.

Neurons were thawed into 10 ml advanced DMEM/F12, centrifuged for 5 minutes at 200 g, resuspended in NMM +10 µM Rho Kinase inhibitor Y27632 (RI) + 20 ng/ml bFGF and plated in one Geltrex-coated well of a 6 well plate. The next day the medium was replaced with 2 ml NMM. Final plating of the neurons on poly-L-ornithin/laminin coated ibidi 96 well plates was performed at day 35. Cells were washed with PBS and 0.5 ml Accutase was applied to the cells; after 10 minutes incubation cells were brought to suspension by pipetting up and down 3 times in the Accutase and transferred to 10 ml advanced DMEM/F12. Cells were centrifuged

for 5 minutes at 200 g and resuspended in NMM + 10 µM RI. Medium was replaced with NMM the next day and from then on every 2-3 days.

Tables: Reagents for cortical neuron differentiation (modified from Shi et al).

Neural Maintenance media (NMM)			
**Product Name**	**Supplier**	**Catalog number**	**Concentration**
DMEM:F12	Thermo Fisher	21331-020	0.5 x
Neurobasal	Thermo Fisher	1103049	0.5 x
Insulin	Sigma	I9278	2.5 µg/ml
Non essential amino acids	Thermo Fisher	11140035	0.5 x
Sodium Pyruvate	Sigma	S8636	50 µM
N2 supplement	Thermo Fisher	17502048	0.5 x
B27 supplement	Thermo Fisher	17504044	0.5 x
Glutamax	Thermo Fisher	17504044	200 µM
2-mercaptoethanol	Thermo Fisher	31350010	50 µM
**Neuronal Induction Medium (NIM)**			
NMM	self mixed		1 x
SB431542	Medchem	MCE-HY-10431	10 µM
Dorsomorphin	Tocris	3093	1 µM

Further Materials		
Essential 8 medium	Thermo Fisher	A1517001
Geltrex	Thermo Fisher	A1413302
EDTA	Thermo Fisher	15575020
Accutase	Thermo Fisher	A1110501
PBS	Bio Concept	3-05F29-I
advanced DMEM/F12	Thermo Fisher	12634010
Dispase2	Sigma	D4693
FGF2	PeproTech	100-18B
Rho-associated kinase inhibitor Y27632	Medchem	MCE-HY-10583
Poly-L-Ornithin	Sigma	P4957
Laminin521	Thermo Fisher	A29249
6 well tc plates	Greiner	657 160
96 well µ plates	ibidi	89621

2. Proliferation inhibition and Maturation (MAT) protocol

Neurons were differentiated via dual SMAD inhibition as described above. On day 35 the final plating was done on poly-D-lysine/laminin coated 96-well imaging plates and cells were kept in NMM until day 45. On day 45 half of the medium was replaced with proliferation inhibition medium and on day 49 again half of the medium was replaced with proliferation inhibition medium. From day 53 onwards half of the medium was replaced every 3-4 days with maturation medium and cells were assayed on day 70.

Tables: Reagents for the proliferation inhibition and maturation protocol

**Proliferation Inhibition Medium**			
**Product Name**	**Supplier**	**Catalog number**	**Concentration**
Neurobasal Plus medium	Thermo Fisher	A3582901	1 x
B27 Plus	Thermo Fisher	A3582801	1 x
Glutamax 200mM (final 0.5 mM)	Thermo Fisher	35050038	0.5 mM
2-mercaptoethanol	Thermo Fisher	21985023	50 µM
GDNF	Alomone	G-240	20 ng/ml
BDNF	Alomone	B-250	20 ng/ml
Bucladesine (sodium)	MedChemExpress	MCE-HY-B0764	2 mM
DAPT	MedChemExpress	MCE-HY-13027	10 µM
SU-5402	MedChemExpress	MCE-HY-10407	10 µM
Palbociclib	MedChemExpress	MCE-HY-50767	4 µM
Vitamin C	Sigma	A4403	400 nM

**Maturation Medium**			
Neurobasal Plus medium	Thermo Fisher	A3582901	1x
B27 Plus	Thermo Fisher	A3582801	1x
Glutamax	Thermo Fisher	35050038	0.5 mM
2-mercaptoethanol	Thermo Fisher	21985023	50 µM
GDNF	Alomone	G-240	20 ng/ml
BDNF	Alomone	B-250	20 ng/ml
Bucladesine (sodium)	MedChemExpress	MCE-HY-B0764	2 mM
Palbociclib	MedChemExpress	MCE-HY-50767	4 µM
Vitamin C	Sigma	A4403	400 nM

**Further Materials**		
Poly-D-Lysine	Thermo Fisher	A3890401
Laminin521	Thermo Fisher	A29249

3. Gibco NSC protocol

hiPSCs were differentiated to NSCs following the Gibco Neural induction protocol (https://www.thermofisher.com/order/catalog/product/A1647801). Briefly, hiPSCs were maintained in StemFlex medium on Geltrex-coated 6 well plates and passaged as small cell clusters using 0.5 mM EDTA. On the day of induction hiPSCs, which had reached a confluency of around 70%, were detached with 0.5 mM EDTA and subsequently dissociated with Accutase and replated in neuronal induction medium at a density of 2.5 $x10^5$ cells/well into a Geltrex-coated 6 well plate where cells were further cultured in neuronal induction medium for the next 7 days. Following the Gibco protocol, NSCs were harvested and expanded on day 7 of neural induction. Cells were split with Accutase and seeded at a density of 200'000 cells/ well of a 6 well plate in neural expansion medium +RI until the 5th passage, thereafter cells were maintained in neural expansion medium without addition of RI. After 8-10 passages, cells were split into Geltrex-coated 8 well ibidi slides and fixed 2 days after splitting, when cells reached a confluency of around 70%.

Tables: Reagents for the GIBCO NSC protocol

**Neural Induction Medium**			
**Product Name**	**Supplier**	**Catalog number**	**Concentration**
Neurobasal	Thermo Fisher	21103049	1x
GIBCO® Neural Induction Supplement	Thermo Fisher	A1647801	1x
Neural Expansion Medium			
Neurobasal	Thermo Fisher	21103049	0.5x
advanced DMEM/F12	Thermo Fisher	12634010	0.5x
GIBCO® Neural Induction Supplement	Thermo Fisher	A1647801	1x
**Materials**			
Gibco® PSC Neural Induction Medium	Thermo Fisher	A1647801	
EDTA	Thermo Fisher	15575020	
StemPro® Accutase®	Thermo Fisher	A11105	
Rho-associated kinase inhibitor Y27632	Medchem	MCE-HY-10583	
Poly-Ornithin	Sigma	P4957	
Laminin521	Thermo Fisher	A29249	
6 well tc plates	Greiner	657160	
8 well µ plates	ibidi	80801	

4. i3 NGN2 cortical neuron protocol (Fernandopulle et al. 2018)

Human induced pluripotent stem cells (hiPSCs) were differentiated into glutamatergic neurons of cortical subtype by transient overexpression of the transcription factor Neurogenin 2 (NGN2) as described previously, with minor modifications (Fernandopulle et al. 2018). The hiPSC line WTC11 containing a stably integrated NGN2 transgene under a tetracycline-inducible promoter (NWTC11.G3-WT) was obtained from the Gan laboratory at the Gladstone Institute of Neurological Disease (now Weill Cornell).

hiPSCs were cultured and maintained in an undifferentiated state during their expansion. Throughout the protocol, Matrigel-coated tissue culture plates and dishes were used, and the cells were cultured in Essential 8 medium (E8). The cells were passaged with Accutase when they reached approximately 80% confluency and replated in E8 medium supplemented with RI to prevent death of single cells.

For neuronal induction hiPSCs were dissociated with Accutase, counted and 2×10^6 cells were resuspended in 10 ml induction medium (IM) + RI and replated in a Matrigel coated 10 cm tissue culture dish. The following two days a full medium change was performed with IM without RI supplementation. Cultures composed of healthy cells with neurites were dissociated with Accutase and frozen as a masterbatch for future experiments.

For experimental use, the cells were thawed and resuspended in cortical neuron culture medium (CM) into polyethyleneimine (PEI), borate and laminin coated imaging plates for further maturation. Half-medium changes with CM were made every four days, and experiment endpoints were at day 12 and day 20 after NGN2 induction.

Tables: Reagents NGN2 i3 cortical neurons

**Induction Medium (IM)**			
**Product Name**	**Supplier**	**Catalog number**	**Concentration**
DMEM/F12, HEPES	Thermo Fisher	11330032	1x
N2 supplement	Thermo Fisher	17502048	1x
Non-essential amino acids (NEAA)	Thermo Fisher	11140050	1x
L-glutamine	Thermo Fisher	25030081	200 µM
Doxycycline	Sigma	D9891	2 µg/ml
Rho-associated kinase inhibitor Y27632	Tocris	1254	10 µM
**Cortical Neuron Culture Medium (CM)**			
**Product Name**	**Supplier**	**Catalog number**	**Amount per 50 mL**
BrainPhys neuronal medium	STEMCELL Technologies	05790	1x
B27 supplement	Thermo Fisher	17504044	1x
BDNF	PeproTech	450-02	10 ng/ml
NT-3	PeproTech	450-03	10 ng/ml
Laminin (mouse)	Thermo Fisher	23017015	200 ng/ml
**Further Materials**			
Essential 8 medium	Thermo Fisher	A1517001	
Accutase	Thermo Fisher	A1110501	
PEI	Sigma	03880	
Borate buffer	Thermo Fisher	28341	
96 well µ plates	ibidi	89621	
Matrigel, hESC-qualified	Corning	354277	

5. Cerebral organoid protocol

Cerebral organoids were generated using a previously described protocol (Lancaster and Knoblich 2014), with minor modifications. The hiPSCs were cultured on Geltrex in StemFlex medium and passaged as small cell clusters using 0.5 mM EDTA. At day 0, hiPSCs were detached with 0.5 mM EDTA and subsequently dissociated with Accutase for embryoid body formation (EB). Single cells were plated at a density of 4500 cells/ well in an ultra-low attachment 96-well plate. Cells were maintained in StemFlex medium supplemented with 4 ng/ml bFGF and 50 µM RI. On day 2, medium was replaced with StemFlex medium supplemented with bFGF only.

On day 4 of differentiation Neural induction was initiated, when EBs reached a diameter of 350-600 µm. The 96-well plates containing the EBs were washed twice with 200 µl neural induction medium (NIM) and cultured in 150 µl NIM. Media changes were performed every 2 days. After 4 days, the EBs were expected to form a primitive neuroepithelium, which causes a brightening of the smooth edges of the EBs. Once the neuroepithelium was formed (usually day 8), each EB was embedded in 25 µl Matrigel and stationary differentiation in 60 mm culture dishes was started. Each 60 mm dish contained 16-20 EBs in 5 ml differentiation medium without vitamin A.

Stationary culture was kept in the incubator for 4 days with a full media change after 48 h. After the stationary culture phase, excess Matrigel was removed and media was changed to 5 ml differentiation media containing vitamin A. Cerebral organoids were differentiated up to day 45 on an orbital shaker at 37° C and 5 % CO2 (shaker orbit: 19 mm; shaking speed 74 rpm). Full media changes were performed 3 x per week.

Tables: Reagents for cerebral organoid protocol

**Embryoid Body (EB) Medium**			
**Product Name**	**Supplier**	**Catalog number**	**Concentration**
bFGF	Peprotech	100-18B	4 ng/ml
StemFlex	Thermo Fisher	A3349401	1 x
(Rho-associated kinase inhibitor Y27632)	Lucerna Chem	MCE-HY-10583	50 µM
**Neural Induction medium**			
DMEM-F12	Thermo Fisher	21331046	1 x
N2 Supplement	Thermo Fisher	17502048	1 %
GlutaMAX™	Thermo Fisher	7001576	1 %
Heparin	Sigma	H3149	1 µg/ml
MEM-Non essential amino acids	Thermo Fisher	7001634	1 %

Differentiation Medium stationary culture			
2-Mercaptoethanol	Thermo Fisher	31350-10	28.5 µM
B27 without VitA	Thermo Fisher	7002266	1%
DMEM-F12	Thermo Fisher	21331046	50%
GlutaMAX™	Thermo Fisher	7001576	1%
Insulin, human recombinant	Sigma	I9278	2.5 µg/ml
MEM-Non essential amino acids	Thermo Fisher	7001634	0.5%
N2 Supplement	Thermo Fisher	17502048	0.5%
Neurobasal medium	Thermo Fisher	1103049	50%
Penicillin-Streptomycin	Thermo Fisher	7002242	1%
**Differentiation Medium shaking culture**			
2-Mercaptoethanol	Thermo Fisher	31350-10	28.5 µM
B27 with VitA	Thermo Fisher	17504044	1%
DMEM-F12	Thermo Fisher	21331046	50%
GlutaMAX™	Thermo Fisher	7001576	1%
Insulin, human recombinant	Sigma	I9278	2.5 µg/ml
MEM-Non essential amino acids	Thermo Fisher	7001634	0.5 %
N2 Supplement	Thermo Fisher	17502048	0.5 %
Neurobasal medium	Thermo Fisher	7001626	50 %
Penicillin-Streptomycin	Thermo Fisher	7002242	1 %
**Further Materials**			
StemPro© Accutase	Thermo Fisher	7002268	
UltraPure™ 0.5M EDTA	Thermo Fisher	15575-038	
CORNING®MATRIGEL®HESC-QUALIFIED MATRIX	Corning	FAL356277	
Orbital shaker	Thermofisher	88881101	
96 Well plate ultra-low adhesion	Corning	CLS7007-24EA	

6. Dopaminergic neuron protocol

Midbrain patterned dopaminergic neurons were derived following a previously published protocol (Kriks et al. 2011), with minor modifications.

hiPSC cultured in StemMACS iPSC Brew medium were passaged 2 days before induction as small cell clusters using 0.5 mM EDTA into a well of a Geltrex coated tissue culture plate (6 well or 12 well). On the day of induction, the hiPSC need to reach a confluency of more than 90%. Cells were washed with PBS and the neuronal differentiation was started by adding day0 medium. For the first 12 days medium was replaced daily, with changes of basal medium and the factors for midbrain patterning as summarized in tables below. Importantly the basal media was prepared once for the whole experiment, but for optimal performance the factors were added freshly every day. From day 13 onwards the cells were kept in maturation medium with medium changes every 2-3 days. On day 20 the cells were brought to single cell suspension with Accutase and replated to poly-L-ornithin/laminin coated 96 well imaging plates in maturation medium supplemented with 10 µM RI. Medium was replaced the next day with maturation medium. On day 24, neurons were either fixed or treated with Mitomycin C 1 µg/ml for 1 hour to remove proliferation cells, followed by a wash with NB medium and addition of fresh maturation medium. Again, cells were fed every 2-3 days with fresh maturation medium until day 35 when cells were fixed.

Tables: Reagents dopaminergic neurons

**KSR KO DMEM medium**			
**Product Name**	**Supplier**	**Catalog number**	**Concentration**
KO DMEM	Thermo Fisher	10829018	73 %
KO Serum replacement	Thermo Fisher	10828028	15 %
Glutamax	Thermo Fisher	35050061	1 %
Non essential amino acids	Thermo Fisher	11140035	1 %
2-mercaptoethanol	Thermo Fisher	31350010	10 µM
**NNB medium**			
Neurobasal	Thermo Fisher	1103049	97.5 %
N2	Thermo Fisher	17502048	0.5 %
B27	Thermo Fisher	17504044	1 %
Glutamax	Thermo Fisher	35050061	1 %
**NB medium**			
Neurobasal	Thermo Fisher	1103049	97 %
B27	Thermo Fisher	17504044	2 %
Glutamax	Thermo Fisher	35050061	1 %
**Day0 medium**			
KSR KO DMEM	self mixed		
LDN	Medchem	S2618	100 nM

SB431542	Medchem	MCE-HY-10431	10 µM
**Day1+2 medium**			
KSR KO DMEM	self mixed		100 %
LDN	Medchem	S2618	100 nM
SB431542	Medchem	MCE-HY-10431	10 µM
SHH C24II	R&D Systems	1845-SH-025	100 ng/ml
Purmorphamine	Millipore	540220	2 µM
FGF8			100 ng/ml
**Day3+4 medium**			
KSR KO DMEM	self mixed		100 %
LDN	Medchem	S2618	100 nM
SB431542	Medchem	MCE-HY-10431	10 µM
SHH C24II			100 ng/ml
Purmorphamine	540220		2 µM
FGF8			100 ng/ml
CHIR-99021			3 µM
**Day5+6 medium**			
KSR KO DMEM	self mixed		75 %
NNB	self mixed		25 %
LDN	Medchem	S2618	100 nM
SB431542	Medchem	MCE-HY-10431	10 µM
SHH C24II	Bio Techne	1845-SH-100	100 ng/ml
Purmorphamine	Sigma	540220	2 µM
FGF8	Bio Techne	4745-F8-050	100 ng/ml
CHIR-99021	Medchem	HY-10182	3 µM
**Day5+6 medium**			
KSR KO DMEM	selfmixed		75 %
NNB	self mixed		25 %
LDN	Medchem	S2618	100 nM
SB431542	Medchem	MCE-HY-10431	10 µM
SHH C24II	Bio Techne	1845-SH-100	100 ng/ml
Purmorphamine	Sigma	540220	2 µM
FGF8	Bio Techne	4745-F8-050	100 ng/ml
CHIR-99021	Medchem	HY-10182	3 µM
**Day7+8 medium**			
KSR KO DMEM	self mixed		50 %
NNB	self mixed		50 %
LDN	Medchem	S2618	100 nM
CHIR-99021	Medchem	HY-10182	3 µM
**Day9+10 medium**			
KSR KO DMEM	self mixed		25 %
NNB	self mixed		75 %
LDN	Medchem	S2618	100 nM
CHIR-99021	Medchem	HY-10182	3 µM

Day11+12 medium			
NB medium	self mixed		100 %
BDNF	Alomone Labs	B-250	20 ng/ml
GDNF	Alomone Labs	G-240-0.1MG	20 ng/ml
TGFB3	Thermo Fisher	PHG9305	1 ng/ml
DAPT	Medchem	MCE-HY-13027	10 µM
Ascorbic Acid	Sigma	A4403	200 µM
Bucladesine (sodium)	MedChem	MCE-HY-B0764	200 µM
CHIR-99021	Medchem	HY-10182	3 µM
**Day13-19 medium (maturation medium)**			
NB medium	self mixed		100 %
BDNF	Alomone Labs	B-250	20 ng/ml
GDNF	Alomone Labs	G-240-0.1MG	20 ng/ml
TGFB3	Thermo Fisher	PHG9305	1 ng/ml
DAPT	Medchem	MCE-HY-13027	10 µM
Ascorbic Acid	Sigma	A4403	200 µM
Bucladesine (sodium)	MedChem	MCE-HY-B0764	200 µM
**Day20 medium (maturation medium+Rhoki+Laminin)**			
NB medium	self mixed		100 %
BDNF	Alomone Labs	B-250	20 ng/ml
GDNF	Alomone Labs	G-240-0.1MG	20 ng/ml
TGFB3	Thermo Fisher	PHG9305	1 ng/ml
DAPT	Medchem	MCE-HY-13027	10 µM
Ascorbic Acid	Sigma	A4403	200 µM
Bucladesine (sodium)	MedChem	MCE-HY-B0764	200 µM
Rho-associated kinase inhibitor Y27632	Medchem	MCE-HY-10583	10 µM
Laminin 521	Thermo Fisher	A29249	1 µg/ml
**Day21-35 medium (maturation medium+Laminin)**			
NB medium	selfmixed		100 %
BDNF	Alomone Labs	B-250	20 ng/ml
GDNF	Alomone Labs	G-240-0.1MG	20 ng/ml
TGFB3	Thermo Fisher	PHG9305	1 ng/ml
DAPT	Medchem	MCE-HY-13027	10 µM
Ascorbic Acid	Sigma	A4403	200 µM
Bucladesine (sodium)	MedChem	MCE-HY-B0764	200 µM
Laminin 521	Thermo Fisher	A29249	1 µg/ml

Further Materials		
StemMACS iPSC Brew	Miltenyi Biotec	130-107-086
Mitomycin C	Sigma	M4287
EDTA	Thermo Fisher	15575020
Accutase	Thermo Fisher	A1110501
PBS	Bio Concept	3-05F29-I

advanced DMEM/F12	Thermo Fisher	12634010
Rho-associated kinase	Medchem	MCE-HY-10583
Poly-L-Ornithin	Sigma	P4957
Laminin521	Thermo Fisher	A29249
6 and 12 well tc plates	Greiner	
96 well µ plates	ibidi	89621

7. Histology and immunofluorescence (IF) of cerebral organoids

7.1. Adherent cells

Adherent cells (hiPSC, NSCs, neurons and macrophages) were cultured in 96 well µ ibidi plates. They were washed with PBS, fixed with 4 % PFA for 20 minutes followed by PFA quenching with 100 mM glycine and stored in PBS at 4°C until start of the immunofluorescence staining for a maximum of 2 weeks.

Cells were blocked with PBS +0.3 % TritonX +10 % normal donkey serum (NDS). Then primary antibodies were applied overnight in PBS +0.1 %TritonX +2 % NDS. Cells were washed 3x with PBS +0.3 % TritonX and secondary antibodies were applied for 90 minutes in PBS +0.1% Triton X +2 % NDS. Cells were washed 2 x with PBS + 0.3 % TritonX. Then incubated with PBS+ 0.3 % TritonX +Hoechst for 10 minutes, washed with PBS and mounted with ibidi mounting solution.

For detection of cells in S-phase the EDU Click-iT kit (C10337) was used following the manufactures instructions. Before fixation cells were treated for 30 minutes with 10 µM EDU. We proceeded to the Click reaction on the day of fixation, followed by an immunostaining with antibodies as described above.

7.2. cerebral organoids

Organoids were washed in PBS and fixed with 4 % (w/v) paraformaldehyde at RT with fixation times depending on the size and age of the organoids: 10 minutes for 15-day-old organoids, 15-20 minutes for 45-day-old organoids. Once the fixation period elapsed, organoids were quenched with 0.1 M PBS-glycine for 5 minutes. This was followed by three 5-minute washes using 1X PBS only.

In order to prepare for cryosectioning, samples were cryoprotected in 30 % sucrose solution (in PBS) at 4° C overnight. Subsequently, organoids were embedded into O.C.T compound and snap frozen with liquid nitrogen. Embedded frozen organoids were cryosectioned with a cryostat (Cryostar NX50, Thermo scientific) in 16 µm slices and section were mounted on SuperFrost plus slides (Epredia™).

To perform immunofluorescence staining, sections were thawed for 10 min, PBS washed and treated for 2 h with blocking solution consisting of 5 % BSA, 5% normal goat serum and 0.3% Triton-X-100 in PBS. Subsequently blocked sections were washed with PBS once and incubated with primary antibodies diluted in 1 % BSA ,1 % NGS, 0.1 % Triton-X-100 in PBS at 4° C overnight. Sections were washed three times with PBS and incubated with secondary antibodies diluted in in 1 % BSA ,1 % NGS, 0.1 % Triton-X-100 in PBS for 2 h in the dark at RT. Antibody-stained slides were washed at least three times with PBS followed by a 10 min nuclei counterstain with Hoechst. After a final wash with PBS, slides were mounted with ProLong™ Gold antifade mounting solution.

Table: Reagents for histology and immunofluorescent staining

Antibodies used in 2D and 3D cultures				
**Reagent**	**Supplier**	**Catalog number**	**Dilution 2D**	**Dilution 3D**
AF 488, Goat anti-mouse IgG1	Thermo Fisher	A21121	1:500	1:1000
AF 546, Goat anti-mouse IgG2a	Thermo Fisher	A21133	1:500	1:1000
AF 555, Goat anti-chicken IgY	Thermo Fisher	A32932	1:500	1:1000
AF 568, Goat anti-mouse IgG1	Thermo Fisher	A21124	1:500	1:1000
AF 647, Goat anti-Rabbit	Thermo Fisher	A21245	1:500	1:1000
Chicken anti-TH	Aves	TYH-0020	1:1000	-
Chicken anti-MAP2 A, B+C	abcam	Ab5392	1:1000	-
Mouse anti-ARL13B (IgG2a)	Biolegend	857601	1:500	1:500
Mouse anti-CEP164 (IgG2a)	Santa Cruz	sc-515403	1:200	1:200
Mouse anti-MAP2A+B (IgG1)	Millipore	MAB378	1:300	1:300
Mouse anti-TUBB3 (IgG2a)	Biolegend	801202	1:300	-
Mouse anti-Nestin (IgG1)	Millipore	MAB5326	1:300	1:300
Mouse anti-PCNT (IgG1)	abcam	ab28144	1:1000	1:1000
Mouse anti-PAX6 (IgG1)	Thermo Fisher	14-9914-80	1:100	-
Rabbit anti-AC3	abcam	ab125093	1:200	1:200
Rabbit anti-ARL13B	Proteintech	17711-1-AP	1:200	1:500
Rabbit anti-B4spectrin	Thermo Fisher	PA5-62972	1:500	-
Rabbit anti-FOXG1	abcam	ab18259	1:500	-
Rabbit anti-GPR161	Proteintech	13398-1-AP	1:200	1:200
Rabbit anti-INPP5E	Proteintech	17797	1:100	1:100
Rabbit anti-PAX6	Biolegend	Poly19013	1:300	1:300

Further Materials		
**Product Name**	**Supplier**	**Catalog number**
Gycine	Sigma	G8898
Normal donkey serum	Sigma	S30-M
Normal goat serum	Thermo Fisher	31873
ibidi mounting medium	ibidi	50001
Bovine Serum Albumin	Sigma	A3294
Hoechst 33342	Thermo Fisher	62249
O.C.T Compound	Tissue Tek	62550-01
Pierce™ 16% Formaldehyde	Thermo Fisher	28908
ProLong™ Gold antifade	Invitrogen™	P36930
Sucrose	Sigma	S7903
Triton-X-100	Panreac	A4975
NucBlue™ (Hoechst33342)	Invitrogen™	CAT# R37606
EDU Click-iT kit Alexa 488	Thermo Fisher	C10337

Supplementary References

Fernandopulle, Michael S.; Prestil, Ryan; Grunseich, Christopher; Wang, Chao; Gan, Li; Ward, Michael E. (2018): Transcription Factor-Mediated Differentiation of Human iPSCs into Neurons. In *Current protocols in cell biology* 79 (1), e51. DOI: 10.1002/cpcb.51.

Kriks, Sonja; Shim, Jae-Won; Piao, Jinghua; Ganat, Yosif M.; Wakeman, Dustin R.; Xie, Zhong et al. (2011): Dopamine neurons derived from human ES cells efficiently engraft in animal models of Parkinson's disease. In *Nature* 480 (7378), pp. 547–551. DOI: 10.1038/nature10648.

Lancaster, Madeline A.; Knoblich, Juergen A. (2014): Generation of cerebral organoids from human pluripotent stem cells. In *Nature protocols* 9 (10), pp. 2329–2340. DOI: 10.1038/nprot.2014.158.

Shi, Yichen; Kirwan, Peter; Livesey, Frederick J. (2012): Directed differentiation of human pluripotent stem cells to cerebral cortex neurons and neural networks. In *Nature protocols* 7 (10), pp. 1836–1846. DOI: 10.1038/nprot.2012.116.
